# Unraveling the Genetic Basis of Fertility Restoration for Cytoplasmic Male Sterile Line *WNJ01A* Originated From *Brassica juncea* in *Brassica napus*

**DOI:** 10.3389/fpls.2021.721980

**Published:** 2021-08-31

**Authors:** Qian Yang, Xiaoyi Nong, Jize Xu, Fan Huang, Fang Wang, Jiangsheng Wu, Chunyu Zhang, Chao Liu

**Affiliations:** College of Plant Science and Technology, Huazhong Agricultural University, Wuhan, China

**Keywords:** *Brassica napus*, cytoplasmic male sterility, fertility restorer, map-based cloning, candidate gene, transcriptome

## Abstract

Crosses that lead to heterosis have been widely used in the rapeseed (*Brassica napus* L.) industry. Cytoplasmic male sterility (CMS)/restorer-of-fertility (*Rf*) systems represent one of the most useful tools for rapeseed production. Several CMS types and their restorer lines have been identified in rapeseed, but there are few studies on the mechanisms underlying fertility restoration. Here, we performed morphological observation, map-based cloning, and transcriptomic analysis of the F_2_ population developed by crossing the CMS line *WNJ01A* with its restorer line *Hui01*. Paraffin-embedded sections showed that the sporogenous cell stage was the critical pollen degeneration period, with major sporogenous cells displaying loose and irregular arrangement in sterile anthers. Most mitochondrial electron transport chain (mtETC) complex genes were upregulated in fertile compared to sterile buds. Using bulked segregant analysis (BSA)-seq to analyze mixed DNA pools from sterile and fertile F_2_ buds, respectively, we identified a 6.25 Mb candidate interval where *Rfw* is located. Using map-based cloning experiments combined with bacterial artificial chromosome (BAC) clone sequencing, the candidate interval was reduced to 99.75 kb and two pentatricopeptide repeat (*PPR*) genes were found among 28 predicted genes in this interval. Transcriptome sequencing showed that there were 1679 DEGs (1023 upregulated and 656 downregulated) in fertile compared to sterile F_2_ buds. The upregulated differentially expressed genes (DEGs) were enriched in the Kyoto Encyclopedia of Genes and Genomes (KEGG) lysine degradation pathway and phenylalanine metabolism, and the downregulated DEGs were enriched in cutin, suberine, and wax biosynthesis. Furthermore, 44 DEGs were involved in pollen and anther development, such as tapetum, microspores, and pollen wall development. All of them were upregulated except a few such as *POE1* genes (which encode Pollen Ole e I allergen and extensin family proteins). There were 261 specifically expressed DEGs (9 and 252 in sterile and fertile buds, respectively). Regarding the fertile bud-specific upregulated DEGs, the ubiquitin–proteasome pathway was enriched. The top four hub genes in the protein–protein interaction network (BnaA09g56400D, BnaA10g18210D, BnaA10g18220D, and BnaC09g41740D) encode RAD23d proteins, which deliver ubiquitinated substrates to the 26S proteasome. These findings provide evidence on the pathways regulated by *Rfw* and improve our understanding of fertility restoration.

## Introduction

Rapeseed (*Brassica napus* L.) is one of the most important oil crops worldwide. Crosses that lead to heterosis, involving significantly increased seed yield, have been widely used for rapeseed production in the past decades (Morrison et al., 2016; Shen et al., 2017). Cytoplasmic male sterility (CMS) combined with its maintainer line and restorer line is a highly valuable resource to produce hybrid seeds of rapeseed (Li et al., 2015).

Plant CMS is generally caused by new chimeric open reading frames (ORFs) in the mitochondrial genome, and they are usually generated through mitochondrial genome rearrangement (Tang H. et al., 2017). These novel ORFs are usually homologous or co-transcribed with the genes encoding proteins of the mitochondrial electron transport chain (mtETC) complexes or ATP synthase, so these systems do not function normally (Bohra et al., 2016). *Restorer-of-fertility* (*Rf*) genes in the nuclear genomes of the restorer lines can downregulate the CMS genes to reverse male sterility. Several *Rf* genes, which encode diverse functional proteins, have been identified. For instance, maize *Rf2* and *Rf4* encode an aldehyde dehydrogenase and a bHLH transcription factor (TF) for T-CMS and C-CMS, respectively (Liu and Schnable, 2002); rice *RF17* and *RF2* encode an acetyl-carrier protein and a glycine-rich protein for CW-CMS and LD-CMS, respectively (Fujii and Toriyama, 2009); and sugar beet *Rf1* encodes a putative M48 family peptidase for Owen-CMS (Kitazaki et al., 2015). However, most cloned plant *Rf* genes encode pentatricopeptide repeat (PPR)-containing proteins (Kubo et al., 2020). These proteins are RNA-binding factors that participate in mRNA processing after transport to a mitochondrion (Schmitz-Linneweber and Small, 2008). During the fertility restoration of CMS, they downregulate sterility genes by cleaving their transcripts or inhibiting their translation (Gaborieau et al., 2016).

In *B. napus*, there are a variety of CMS systems, such as *pol*, *ogu*, *nap*, *hau, Nsa*, and *inap*. Among them, the *pol* and *ogu* CMS lines are the most widely used to produce hybrid seeds. Regarding *pol* CMS, a chimeric *ORF*, *orf224*, is co-transcribed with *atp6* and causes male sterility (Singh and Brown, 1993; L’Homme et al., 1997; Liu et al., 2016). Its restorer gene, *Rfp*, encodes a mitochondria-targeted PPR protein that downregulates *orf224* (Liu et al., 2016). *Ogu* CMS originated in *Raphanus sativus* and was introduced into *B. napus*. Regarding *ogu* CMS, *orf138* is considered to be the gene that causes male sterility (Bonhomme et al., 1992). Its restorer gene *Rfo* from *R*. *sativus* encodes a PPR protein that downregulates orf138 protein at the translation level (Uyttewaal et al., 2008). Another *B. napus* CMS system, *nap*, is rarely used to produce hybrid seeds because the sterility is easily affected by temperature change in most cases. In this system, *orf222* is the key sterility gene, and it is co-transcribed with both *orf139* and an exon of a trans-spliced gene, *nad5c* (L’Homme et al., 1997). Its restorer gene, *Rfn*, on chromosome A09 of *B. napus*, encodes a PPR protein. However, the accumulation of *orf222* transcripts was not obviously changed in the restorer line or in transgenic fertility-restored lines compared to the *nap* CMS line (Liu et al., 2017).

In recent years, new CMS systems from *Brassica* or related species have been discovered and introduced into *B. napus*. The *hau* CMS system originated from *Brassica juncea* and its sterility gene, *orf288*, encodes a cytotoxic protein that causes aborted pollen development (Wan et al., 2008; Jing et al., 2012). *orf288* transcription was upregulated in the male-sterile line compared to the restorer line, but there was no significant difference at the protein level (Heng et al., 2018). A recent research reported that the restorer gene *Rfh* of *hau* CMS has been cloned (Wang H. et al., 2021). *Nsa* CMS is a novel *B*. *napus* CMS system derived from the cytoplasm of *Sinapis arvensis*, and its restorer line *NR1* was developed from a disomic alien addition line of *B. napus* and *S. arvensis* (Wei et al., 2010). Three *ORFs* (*orf224*, *orf309*, and *orf346*), which possess chimeric and transmembrane structures, are the candidate sterility genes of *Nsa* CMS (Sang et al., 2019). It was further confirmed that *ORF346* is the key gene for pollen abortion in *Nsa* CMS (Sang et al., 2021). Another CMS system, *inap* CMS, was obtained via backcross of the somatic hybrid of *Isatis indigotica* (Chinese woad) and *B*. *napus*, with *B*. *napus* as the recurrent parent (Kang et al., 2017). A restorer line carrying a dominant *Rf* gene was successfully developed for *inap* CMS (Li et al., 2019). Further research is required to isolate their restorer genes and use them in rapeseed breeding.

Anther and pollen development are extremely complex processes involving many genes and pathways. Therefore, transcriptome sequencing has been applied in many crops as an effective tool to study global transcription networks to elucidate underlying mechanisms. To elucidate the genome-wide molecular mechanisms underlying *Brassica* CMS and fertility restoration, transcriptomic profiling using RNA-seq has been used. For example, transcriptomic analysis of *pol* CMS indicated that the energy deficiency caused by *orf224/atp6* may downregulate many genes involved in pollen development via nuclear–mitochondrial interactions (An et al., 2014). Through multi-omics joint analysis and yeast two-hybrid assay, seven *Rfp* interacting proteins related to RNA editing, anther and tapetum development, and five *orf224* interacting proteins participating in the electronic respiratory transmission chain, anther development and oxidative phosphorylation were obtained and further verified (Wang B. et al., 2021). In *ogu* CMS, sterility may be caused by delayed tapetum degradation, as genes related to tapetum programmed cell death were downregulated compared to in the maintainer line. Additionally, sporopollenin biosynthesis and its key genes were both inhibited in *ogu* CMS (Xing et al., 2018; Lin et al., 2019). Another transcriptomics study on o*gu* CMS, *pol* CMS, and their shared maintainer line reported that most of the DEGs that were downregulated in both CMS lines were involved in pollen development, carbon metabolism, lipid metabolism, the tricarboxylic acid (TCA) cycle, and oxidative phosphorylation (Wei et al., 2020).

Currently, many hybrid rapeseed cultivars are bred using the *pol* CMS system in China. Thus, it is necessary to develop new stable CMS systems (with restorer lines) to solve the issue of cytoplasmic simplification (i.e., most of the sterile cytoplasm of current commercial hybrids belong to a single type) to avoid risks to the rapeseed industry. We previously developed a novel type of CMS, *WNJ01A*, from a natural male-sterile *B. juncea* mutant. It was introduced into *B. napus* and displayed a stable and complete pollen-abortion phenotype. Recently, its restorer line, *Hui01*, was obtained through distant hybridization between *WNJ01A* CMS and *B. rapa*. Here, we aimed to finely map and isolate the candidate restorer gene, designated *Restorer of fertility for WNJ01A* (*Rfw*). We also aimed to conduct a transcriptomic analysis to identify the genes and pathways involved in male sterility and fertility restoration. The results will facilitate understanding of the molecular mechanisms of *WNJ01A* CMS and its fertility restoration, and they will also provide a foundation for its use in breeding.

## Materials and Methods

### Plant Materials

We previously developed the *WJS1A* CMS line from a natural *B. juncea* mutant. To obtain *WNJ01A* CMS, the sterile cytoplasm was introduced into *B. napus* cv. Huashuang4 by backcrossing for more than 10 generations. Subsequently, we crossed *WNJ01A* CMS (female parent) with *B. rapa* ssp. *pekinensis* via distant hybridization. We then searched for fertile plants in their progenies after continuous selfing, the fertile progeny lines were obtained and it was further confirmed that they could reverse the sterility in *WNJ01A* CMS by test crossing. One restorer line with the best ability of restoring fertility was designated *Hui01*.

### Genetic Analysis of Fertility Restoration for *WNJ01A*

*WNJ01A* (female parent) was crossed with restorer line *Hui01* (male parent) to produce the F_1_ population and then the F_2_ population was generated via self-pollination of F_1_ plants. The fertility of each plant was determined by assessing at least five flowers per plant a minimum of three difference times during the flowering period, following a previously described method (Liu et al., 2012).

### Cytological Characterization of Fertility Restoration for *WNJ01A*

Fresh fertile and sterile floral buds (0–8 mm) at various developmental stages from F_2_ plants were collected and fixed in FAA fixative (3.7% formaldehyde, 50% ethanol, and 5.0% acetic acid) and processed as previously described (Ning et al., 2019). Paraffin-embedded sections were assessed using the *Arabidopsis thaliana* classification criteria for the anther development period (Chang F. et al., 2011).

### Whole-Genome Resequencing of Bulked DNA Pools

To determine the genomic region containing *Rfw*, 37 extremely fertile plants and 38 extremely sterile plants were selected from the F_2_ population. Genomic DNA was extracted from 100 mg fresh leaves from each plant and purified using a DNA Secure Plant Kit (DP320; TIANGEN, China). A NanoDrop 2000 spectrophotometer (Thermo Fisher Scientific, United States) was used to determine the DNA concentration and quality. Next, 80 ng DNA per plant were mixed to construct the F- and S-pools. A HiSeq 3000 PE150 sequencing platform (Illumina, United States) was used to perform high-throughput library construction and sequencing for the S- and F-pools.

Bulked segregant analysis (BSA)-seq, involving the abovementioned clean genome resequencing data and using the reference genome *B. napus* cv. Darmor-bzh (Chalhoub et al., 2014), was conducted as previously described (Tang Q. et al., 2017; Su et al., 2020). To locate the *Rfw* gene, we calculated the proportion of reads in the F- and S-pools with single-nucleotide polymorphism (SNP)/insertion/deletion (InDel) sites that were distinct from the of *Darmor-bzh* reference reads (i.e., SNP/InDel-index) and the difference in this index between the F- and S-pools [i.e., Δ(SNP/InDel-index)]. To eliminate low-quality SNP/InDels, we discarded all loci with an SNP/InDel-index < 0.3 (Takagi et al., 2013). Using the sliding window method (with a 1 Mb window size and 100 kb increment), the average index values of loci were calculated. The SNP/InDel-index of the F- and S-pools and the corresponding Δ(SNP/InDel-index) in the sliding window were used to construct SNP/InDel-index plots.

### Map-Based Cloning of *Rfw*

To develop the set of molecular markers, the InDel polymorphism sites detected during genome resequencing in the candidate interval, simple sequence repeats (SSR) sites in this interval, and publicly available molecular markers in this interval were used. The molecular markers were verified in a mixed DNA pool from fertile and sterile plants, with a small population being used to verify the linkage between the molecular markers and fertility restoration. All primer sequences of linked molecular markers are given in Supplementary Table 1. Next, 4454 sterile F_2_ plants were subjected to map-based cloning experiments to finely map *Rfw*, based on the numbers of recombinant plants and the known physical locations of a set of molecular markers.

### Bacterial Artificial Chromosome (BAC) Screening, Sequencing, and Candidate Gene Prediction

To obtain the candidate region sequence, we constructed a BAC library (using CopyControl^TM^ pCC1BAC^TM^ Vector; EPICENTRE, United States) for the restorer line *Hui01*. *Hin*dIII restriction enzyme was used to digest the genomic DNA and then 90–120 kb DNA fragments were selected by pulse-field electrophoresis and purified. The DNA fragments were ligated into the CopyControl^TM^ pCCIBAC^TM^ Vectors in the library kit. The library contained about 40,000 clones with 90–120 kb DNA insert size, representing about 5 × *B. napus* genome. After batch transformation of EPI300 *E. coli*, the bacterial suspension was evenly smeared onto LB medium containing 12.5 μg/mL chloramphenicol. Thereafter, the strains were grown and then eluted in a sterile centrifuge tube containing chloramphenicol before further propagation. We mixed sets of 1000 single clones to create 40 pools. Next, the BAC plasmids in each pool were extracted using a Large-Construct Kit (QIAGEN, United States). A molecular marker (TY21) that co-segregated with fertility restoration was used to screen for pools containing a positive clone. Finally, the molecular markers flanking the finely mapped candidate interval were used to analyze the physical location of the interval in the positive clones, which underwent high-throughput sequencing using Illumina NovaSeq (Illumina, United States) and Oxford Nanopore Technologies (ONT; United Kingdom).

Four reference genomes containing A subgenomes (*B. rapa* cv. chiifu-401: AA; *B. napus* cv. Darmor-bzh and Zhongshuang11: AACC; *B. juncea* var. *tumida*: AABB) were used to conduct a collinearity analysis of the candidate *Rfw* genes in the finely mapped *Rfw* region. The genes in the candidate interval of *Hui01* were analyzed and predicted through the Fgenesh gene-finder^1^. The gene coding sequence (CDS) was compared and analyzed with ClustalX.

### Analysis of Mitochondrial Gene Transcripts Using RT-qPCR

Annotation information of the *B. juncea* var. *tumida* and *jiangpu* mitochondrial genomes (accession nos. KJ461445 and JF920288, respectively) were obtained from the National Center for Biotechnology Information (NCBI) (Chang S. et al., 2011; Zhao et al., 2016). We selected 32 known mitochondrial genes, including genes related to mtETC complex I (NADH dehydrogenase, *nad*), complex III (ubiquinol–cytochrome c reductase, *cob*), complex IV (cytochrome c oxidase, *cox*), complex V (ATP synthase, *atp*), cytochrome c biogenesis, ribosome small subunit, ribosome large subunit, twin-arginine translocation (*tatC*), and intron maturase (*matR*).

Transcriptional levels in the fertile and sterile buds were assessed using RT-qPCR. All genes and primer sequences are detailed in Supplementary Table 2. A RevertAid First Strand cDNA Synthesis Kit (Thermo Fisher Scientific, United States) was used to reverse transcribe the total RNA following the manufacturer’s protocol. A Hieff^®^ qPCR SYBR Green Master Mix (No Rox) kit (YEASEN, China) and a CFX384 Touch Real-Time PCR Detection System (Bio-Rad, United States) were used for analysis of the transcription levels. *Bna.actin7* was used for normalization in RT-qPCR (for normalization corresponding to total RNA levels).

### Transcriptomic Sequencing

Floral buds (0–2 mm) were carefully harvested from fertile and sterile F_2_ plants. Three biological replicates (involving five buds each) were used for the fertile-pool (F-pool; *Rfw1*, *Rfw2*, and *Rfw3*) and three for the sterile-pool (S-pool; *rfw1*, *rfw2*, and *rfw3*). The buds were frozen in liquid nitrogen at once and then stored at –80°C until further processing. Total RNA was extracted using TRIzol (Invitrogen, United States) according to the manufacturer’s instructions. A HiSeq X Ten platform (Illumina, United States) was used for transcriptome sequencing (Biomarker Technologies, China).

### Identification of DEGs and Enrichment Analyses

Differentially expressed genes (DEGs) between fertile and sterile buds were identified with the DESeq R package (1.10.1), using the Benjamini–Hochberg procedure to adjust the generated *P*-values to control the false discovery rate (FDR). DEGs were identified based on adjusted *P* < 0.05, Fragments Per Kilobase of transcript per Million mapped read (FPKM) fold change ≥ 2, and FDR < 0.01.

Next, we used the GOseq R package based on the Wallenius non-central hypergeometric distribution to perform Gene Ontology (GO) enrichment analysis on the DEGs (Young et al., 2010), which accounts for gene length bias in DEGs. We also performed a Kyoto Encyclopedia of Genes and Genomes (KEGG) enrichment analysis using KOBAS software (Mao et al., 2005). The KEGG and GO analyses were performed via the Bioinformatics platform^2^.

### Interaction Analysis

To predict the protein–protein interactions (PPIs) of the DEGs, the DEG sequences were used in a BLASTx search against the genomes of related species that had PPI information in the STRING database^3^. The PPI network (confidence score > 0.7), with KEGG annotations, was visualized using Cytoscape (Shannon et al., 2003). The hub genes were identified using the Cytoscape plugin CytoHubba (Song et al., 2020; Zuo et al., 2020).

## Results

### Morphological Characterization and Genetic Analysis of CMS and Restorer Lines

**FIGURE 1:**
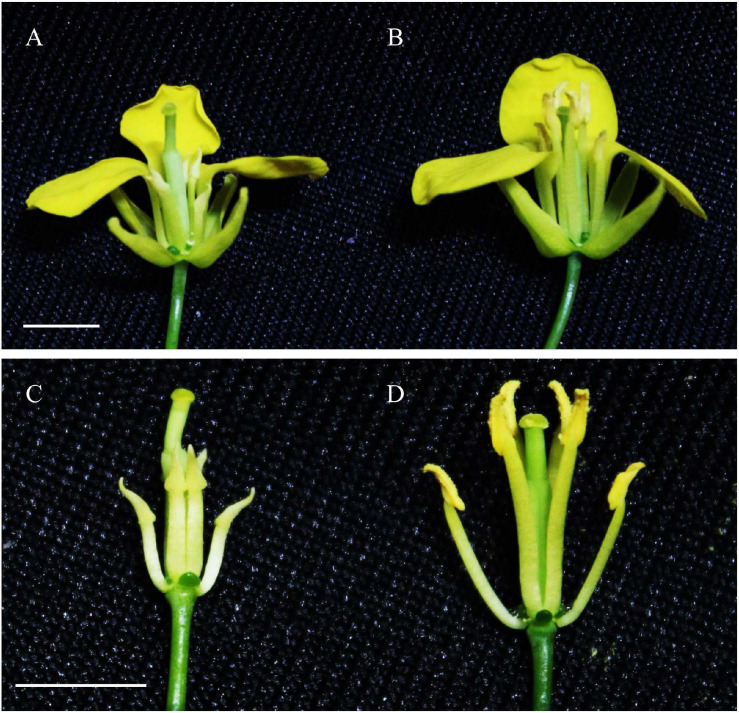
Phenotypic characterization of floral organs. **(A,C)** Sterile flower of CMS line *WNJ01A*. **(B,D)** Fertile flower of restorer line *Hui01*. Bars = 0.5 cm.

The cytoplasm of the CMS line *WNJ01A* originated from a natural male-sterile *B. juncea* mutant. We developed its restorer line *Hui01* via distant hybridization between *WNJ01A* CMS and *B. rapa*. The sepals, petals, and pistils of both the *WNJ01A* and *Hui01* lines exhibited normal development (Figure 1). However, the albino *WNJ01A* anthers were significantly smaller and their filaments were dramatically shorter (Figures 1A,C) compared to the *Hui01* anthers (Figures 1B,D). Across more than 20 generations over 11 years (2010–2020) under various ecological conditions (in spring and winter) in China, *WNJ01A* maintained complete pollen abortion without being affected by temperature or photoperiod.

To elucidate the genetic characteristics of fertility restoration of *WNJ01A* CMS by *Hui01*, the fertility of the F_1_ and F_2_ populations of a *WNJ01A* CMS × *Hui01* cross was evaluated. All F_1_ plants produced normally fertile pollen, indicating that *Hui01* can completely restore the fertility of *WNJ01A* CMS. In the F_2_ population, there were 1606 fertile and 555 sterile plants, which fitted the expected 3:1 ratio (χ_*c*_^2^ = 0.5012, *P* = 0.46). Thus, the fertility restoration of *WNJ01A* CMS is controlled by a dominant gene. It was designated *Restorer of fertility for WNJ01A* (*Rfw*).

### Cytological Characterization of Anther Development

**FIGURE 2:**
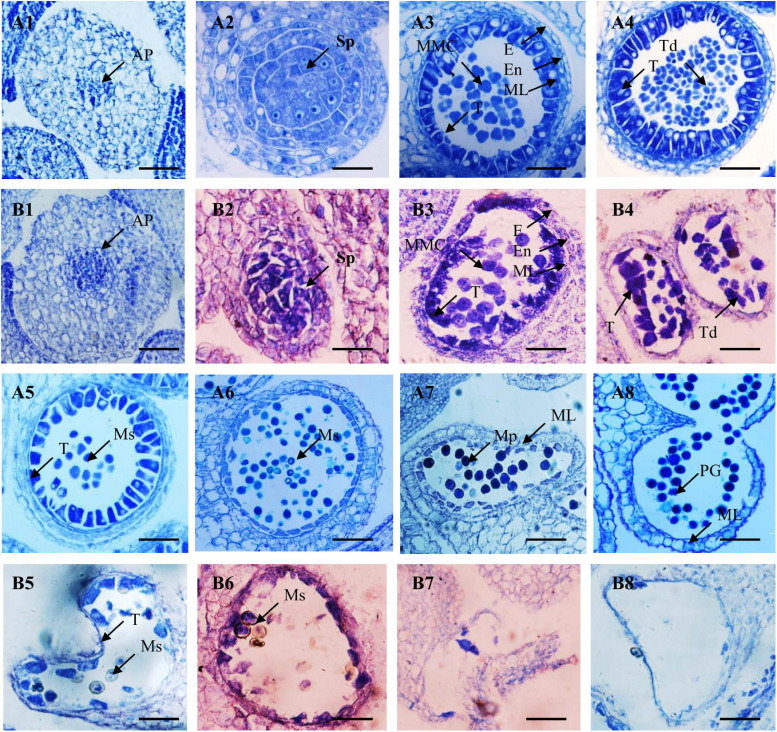
Microstructure of fertile **(A1–A8)** and sterile **(B1–B8)** anthers from F_2_ plants. **(A1,B1)** Anther primordium stage; **(A2,B2)** sporoblast stage; **(A3,B3)** microspore mother cell stage; **(A4,B4)** tetrad stage; **(A5,B5)** early mononuclear stage; **(A6,B6)** mononuclear stage; **(A7,B7)** dinuclear stage; **(A8)** mature microspores; and **(B8)** empty pollen sac. AP, anther primordium; Sp., sporogenous cell; E, epidermis; En, endothecium; ML, middle layer; T, tapetum; MMC, microspore mother cells; Ms, microspore; MP, mature pollen; PG, pollen grain. Bars = 100 μm.

To accurately characterize pollen abortion, paraffin-embedded sections of the buds of fertile and sterile F_2_ plants were observed. At the anther primordia division stage, the cells of anther primordia were dividing, with almost no difference between fertile and sterile anthers (Figures 2A1,B1). However, at the sporogenous cell stage, major sporogenous cells displayed loose and irregular arrangements in sterile anthers, unlike the regular three layers of cells in fertile anthers. The cells in the exothecium, endothecium, and middle layer of the sterile anther walls began to shrink and degenerate. The tapetum cells started to detach from the anther walls and gradually degrade (Figures 2A2,B2). At the microspore mother cell and tetrad stages, microspore mother cells developed normally and then formed numerous tetrads via meiosis in the fertile anthers, and the tapetum cells were regularly arranged. In contrast, the microspore mother cells of the sterile anthers were more scattered, the tetrads were not formed, and the tapetum cells were increasingly abnormal (Figures 2A3,A4,B3,B4).

In the remaining developmental stages (early mononuclear, mononuclear, dinuclear, and mature microspore stages) of the fertile anthers, microspores were released from the tetrads and then many mature pollen grains were found in the anther chambers. The tapetums disintegrated and gradually disappeared, and only the epidermis and fiber layer remained in the pollen sac wall (Figures 2A5–A8). However, in the sterile anthers, the tapetum cells degraded prematurely. Numerous pollen grains became vacuolated and eventually completely degraded, emptying the anther chamber (Figures 2B5–B8).

These results indicate that the sporogenous cell stage is critical for anther development, based on the abnormal *WNJ01A* CMS plants. Equally, this stage is an important stage for *Rfw* to begin to prevent pollen abortion. Furthermore, the anther sterility characteristics of *WNJ01A* CMS were obviously different from the previously studied *pol*, *ogu*, *Nsa*, *hau*, and *inap* CMS *Brassica* systems (Wan et al., 2008; An et al., 2014; Kang et al., 2017; Ding et al., 2018).

### Mapping of *Rfw* Gene

To identify the *Rfw* region, F- and S-pools were constructed by subjecting extremely fertile and sterile F_2_ plants to high-throughput sequencing. A total of 229,832,439 and 167,518,595 reads were obtained from the F- and S-pool, respectively, with a sequencing coverage of 92.14% regarding the *B*. *napus* cv. Darmor-bzh reference genome, and a GC content of 37%. After trimming the raw reads, 82.21 and 60.14 Gb of clean data, respectively, were generated.

**FIGURE 3:**
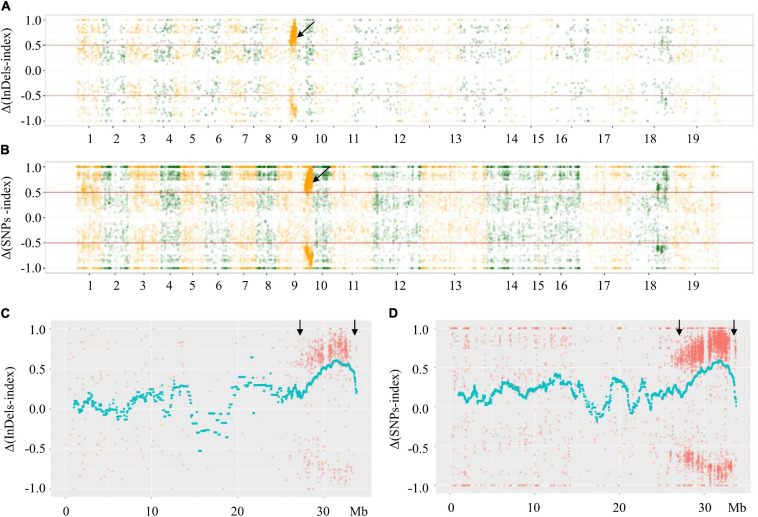
Distributions of Δ(SNPs/InDels-index) across *B. napus* chromosomes. **(A)** Δ(InDels-index) plot obtained by subtraction of F-pool InDel-index from S-pool InDel-index for F_2_ population; **(B)** Δ(SNPs-index) plot obtained by subtraction of F-pool SNP-index from S-pool SNP-index for F_2_ population; **(C)** Δ(InDels-index) on chromosome A09; **(D)** Δ(SNPs-index) on chromosome A09. The *x*-axes show the *B. napus* genome position. The *y*-axes show the index values; red lines in **(A,B)** represent index = | 0.5|. Each spot corresponds to an index value. The fitted curves indicate the average values of Δ(SNPs-index) or Δ(InDels-index) based on sliding window analysis. The black arrows indicate the candidate regions.

Based on comparison with the reference genome, there were 58,610 SNPs and 120,651 InDels in the F- and S-pools. The low-quality SNPs/InDels (SNP/InDel index < 0.3) in both pools were deleted. The indices related to the remaining SNPs/InDels calculated for each pool were plotted onto the 19 *B. napus* chromosomes. Additionally, Δ(SNPs/InDel-index) values (calculated using the sliding window method) were plotted on the 19 *B. napus* chromosomes. The phenotypic difference between the fertile and sterile plants resulted in Δ(SNPs/InDel-index) ≥ 0.5 in a specific genomic locus, which indicates that this region contains the locus underlying the phenotypic difference (i.e., the *Rfw* locus). The significant signal region was only observed on chromosome A09 (not on the other 18 chromosomes) (Figures 3A,B). More specifically, the Δ(SNP/InDel-index) analysis preliminarily mapped *Rfw* to a 27.62–33.87 Mb region (6.25 Mb) on chromosome A09 (Figures 3C,D).

**FIGURE 4:**
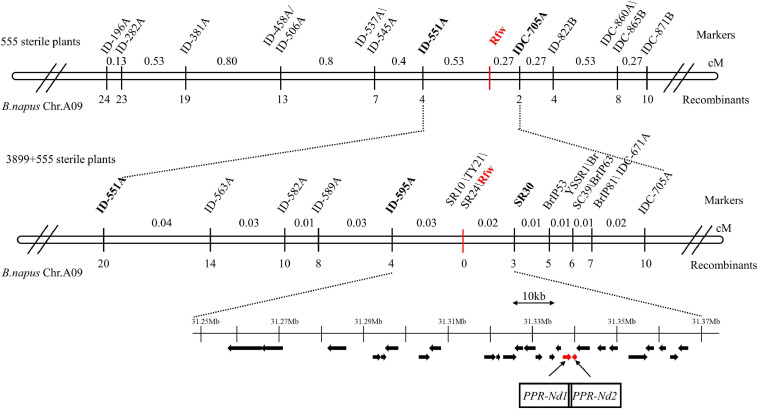
Fine mapping of the *Rfw* locus in *Brassica napus*. The number below each marker indicates the number of recombinant plants (with respect to each given marker and the *Rfw* locus). Black arrows represent predicted genes in the 115.62 kb target region on chromosome A09 of *B. napus*. Red arrows indicate candidate *Rfw* genes.

To verify and narrow down the *Rfw* region, 555 sterile F_2_ plants were used for further mapping. We selected 203 InDel markers in the candidate interval, and 13 of them were found to exhibit polymorphisms between reconstructed F- and S-pools from other fertile and sterile plants, respectively. These 13 markers (Figure 4) were used to assay the 555 sterile plants. We identified 4 and 2 recombinant plants using the markers ID-551A and IDC-705A, respectively, which were the closest markers flanking *Rfw*. Thus, *Rfw* was further mapped to a 30.88–31.98 Mb region on chromosome A09 (Figure 4).

To finely map *Rfw*, 3899 sterile F_2_ plants were further analyzed using the ID-551A and IDC-705A markers, and another 16 and 8 recombinant plants were identified, respectively. Next, we selected 130 markers (81 InDels, 24 SSRs, and 25 publicly available molecular markers) in the 1.1 Mb candidate region, and 16 dominant markers (Figure 4) were found to exhibit polymorphism between reconstructed F- and S-pools. We identified 4 and 3 recombinant plants using ID-595A and SR30, respectively, which were the closest markers flanking *Rfw*. Thus, *Rfw* was located within a genetic distance of 0.05 cM in *B. napus*. Additionally, 3 markers (SR10, TY21, and SR24) co-segregated with the *Rfw* locus in the assayed plants (no recombinant plants) (Figure 4).

### Candidate *Rfw* Genes

To isolate the candidate *Rfw* gene, all the genes (i.e., 26 genes, *BnaA09g45870D*–*BnaA09g46120D*) in the candidate region of the *B. napus* genome (Darmor-bzh and Zhongshuang11) were analyzed. One of these genes is a PPR gene (*BnaA09g46030D*). As the restorer line *Hui01* originated from crossing *WNJ01A* CMS and *B. rapa*, and *WNJ01A* CMS originated from crossing *B. juncea* and *B. napus*, we also analyzed the candidate interval of chromosome A09 in *B. rapa* and *B. juncea*. There were 28 (*Bra026863*–*Bra026890*) and 26 (*BjuA044103*–*BjuA044078*) genes, including two PPR genes (*Bra026882*/*Bra026884* and *BjuA044085*/*BjuA044087*) in the A genome of *B. rapa* cv. chiifu-401 and the AB genome of *B. juncea* var. *tumida*, respectively.

To determine the candidate region sequence, we constructed a BAC library for *Hui01*. Of the nine positive pools, three were randomly selected, and four positive clones were then identified in these pools. The previously identified closest flanking markers (ID-595A and SR30) were used to further confirm the physical location of the inserted fragment in these four positive clones. One clone (M36A7E6) contained the ID-595A locus, and three clones (M16A6E7, M8A5D1, and M8A1H10) contained the SR30 locus (Supplementary Figure 1). Among them, the M36A7E6 and M16A6E7 clones were selected for Illumina HiSeq and PacBio sequencing to obtain the candidate region sequence in *Hui01*.

The DNA insert size of M16A6E7 and M36A7E6 were 99,119 and 101,041 bp and the GC content were 35 and 37%, respectively. The interval between the closest flanking markers ID-595A and SR30 was 99.75 kb, with a 15.87 kb difference between it and the corresponding region (115.62 kb) in the reference genome of Darmor-bzh. Among the 28 predicted genes in this candidate region, there were two PPR genes [*PPR-Nd1:(ORF19)* and *PPR-Nd2:(ORF20)*], which were both only identified in *Hui01* (not in *WNJ01A* CMS). *PPR-Nd1* corresponded to *BnaA09g46030D, Bra026882* and *BjuA044087* while *PPR-Nd2* corresponded to *Bra026884* and *BjuA044085* in the reference genomes. Because the majority of the cloned restorer genes belong to *PPR* family and the other genes except for *PPR-Nd1* and *PPR-Nd2* in this candidate interval have not been verified to be responsible for restoring fertility previously (Supplementary Table 3), the two *PPR* genes were further analyzed.

The CDS length of *PPR-Nd1* in *Hui01* is 1923 bp (with no intron). In comparison, the CDS length is 1794 bp (with a 110-bp intron) in the homolog *BnaA09g46030D* of Darmor-bzh, 1887 bp (with a 29-bp intron) in *BjuA044087* of *B. juncea* var. *tumida*, and 1815 bp (with a 100-bp intron) in *Bra026882* of *B. rapa* cv. chiifu-40. In the 5′ non-PPR domain region of *PPR-Nd1*, there was one InDel (–3 bp) and 24 SNPs compared to its Darmor-bzh homolog *BnaA09g46030D*, along with many InDels and SNPs in the PPR domain region (Supplementary Figure 2). Amino acid sequence alignment showed that there was an amino acid deletion and 10 amino acid substitutions in the 5′ non-PPR domain region that were specific to *PPR-Nd1*. Additionally, there were differences in motifs I and II of the PPR domain. The motif I sequence of *PPR-Nd1* was identical to that of BjuA044087, but BnaA09g46030D and Bra026882 had long-fragment deletions and substitutions. The motif II sequence of *PPR-Nd1* was identical to that of BnaA09g46030D and almost identical to that of Bra026882, while BjuA044087 had two deletions and one long-fragment substitution (Supplementary Figure 3).

*PPR-Nd2* corresponds to homologs *Bra026884* of *B. rapa* and *BjuA044085* of *B. juncea* (none of them have introns). There were a variety of InDels/SNPs in the 5′ region (Supplementary Figure 4) and multiple length reductions and SNPs in the 5′ terminal amino acid sequence (Supplementary Figure 5). As *PPR-Nd2* is not present in the Darmor-bzh genome, PCR amplification from 18 varieties of *B. napus* was carried out, and the gene was found to be present in some of them, such as Yangguang2009 (YG2009). The gene sequence in YG2009 was identical to that in *Hui01*. However, YG2009 appears not to be capable of restoring the fertility of *WNJ01A* CMS (data not shown), which means that this gene is not a candidate *Rfw* gene.

### Analysis of the Expression Levels of Mitochondrial Gene Transcripts

Cytoplasmic male sterility in plants is generally caused by the formation of chimeric ORFs via mitochondrial genome rearrangement, which are usually co-transcribed with known genes or other ORFs (Chen and Liu, 2014). During the fertility restoration of CMS lines, the restorer genes regulate mitochondrial transcription, especially regarding the sterility genes (Li et al., 2018). To identify the mitochondrial genes regulated by *Rfw*, we selected 32 known protein-coding genes (Supplementary Table 2) based on two mitochondrial genomes (NCBI accession no. KJ461445 and JF920288) of *B. juncea* var. *tumida* and *jiangpu*, respectively (Chang S. et al., 2011; Zhao et al., 2016) and assessed their transcriptional levels in fertile and sterile F_2_ buds by RT-qPCR.

**FIGURE 5:**
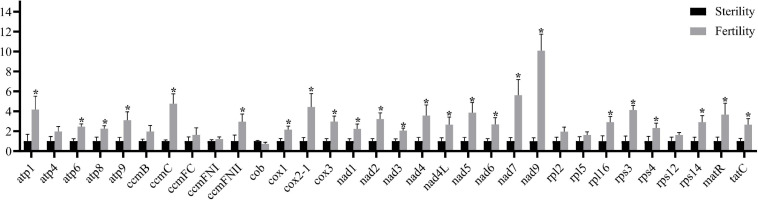
Mitochondrial gene transcriptional levels in fertile and sterile buds (0–2 mm) based on RT-qPCR. The *x*-axis shows the 32 known protein-coding genes from *B. juncea* mitochondrial genomes (NCBI accession nos. KJ461445 and JF920288). *Bna.Actin7* was used as the internal control. Error bars indicate standard deviation (*n* = 3). *P < 0.05.

Most of the 32 known mitochondrial genes showed significant differences in transcription, except for eight genes (Figure 5). Regarding mtETC complexes I, III, IV, and V, nine *nad* genes, three *cox* genes, and four *atp* genes, *ccmC*, and *ccmFN2* were significantly upregulated in fertile buds (and *atp4, ccmB*, *ccmFC*, and *ccmFN1* were non-significantly upregulated while *cob* was non-significantly downregulated). Regarding the ribosome small subunit family, *rps4*, *rps14*, and *rps3* were significantly upregulated (and *rps12* was non-significantly upregulated). Regarding the ribosome large subunit family, only *rpl16* was significantly upregulated (and *rpl2* and *rpl5* were non-significantly upregulated). Both *tatC* and *matR* were also significantly upregulated. These differentially expressed genes may participate in fertility restoration after regulation by *Rfw*.

### DEGs Analysis

To explore the molecular mechanism of *Rfw* regulation of fertility restoration, the transcriptomes of three fertile (*Rfw1*, *Rfw2*, and *Rfw3*) and three sterile (*rfw1*, *rfw2*, and *rfw3*) replicates from the F_2_ population were sequenced. After trimming the raw reads, the number of clean reads in the six samples ranged from 39,052,902 to 61,894,692. A total of 86.43 Gb clean bases was obtained, with each sample having > 11.66 Gb. The GC content was >47% and the Q30 percentage was >90%. Pearson’s correlation coefficients regarding the transcriptional levels among the three sterile bud replicates were ≥0.935 and those among the three fertile bud replicates were ≥0.889 (Supplementary Figure 6). Therefore, the RNA-seq data were suitable for further analysis.

Next, >74% of the clean reads (>70% unique mapped reads and 3.84–4.90% multiple mapped reads) were mapped to the *B. napus* cv. Darmor-bzh reference genome (Table 1).

**TABLE 1:** Sequencing and mapped reads data.

Samples	Replicate	Clean bases	Clean reads	GC content	≥ Q30%	Mapped reads	Unique mapped reads	Multiple map reads
*RFW*	*RFW1*	15,707,725,164	52,560,649	47.14%	91.28%	78,813,689 (74.97%)	74,676,697 (71.04%)	4,136,992 (3.94%)
	*RFW2*	18,482,309,220	61,894,692	47.24%	91.40%	92,665,358 (74.86%)	86,600,145 (69.96%)	6,065,213 (4.90%)
	*RFW3*	11,663,399,684	39,052,902	47.07%	90.65%	58,058,204 (74.33%)	54,879,219 (70.26%)	3,178,985 (4.07%)
*Rfw*	*rfw1*	13,347,442,234	44,679,819	47.26%	90.36%	66,133,402 (74.01%)	62,560,755 (70.01%)	3,572,647 (4.00%)
	*rfw2*	12,497,974,338	41,820,296	47.32%	90.33%	61,937,213 (74.05%)	58,653,225 (70.13%)	3,283,988 (3.93%)
	*rfw3*	14,728,819,074	49,231,140	47.06%	90.04%	72,921,088 (74.06%)	69,142,107 (70.22%)	3,778,981 (3.84%)

We detected 104,348 genes in the RNA-seq data from the six replicates (3229 genes were novel, based on comparison to the reference genome). Subjecting the genes to BLASTx searches against COG, GO, KEGG, Swiss-Prot, eggNOG, and NR databases resulted in 102,843 annotated genes, of which 29253 (28.44%), 78382 (76.22%), 32214 (31.32%), 65560 (63.75%), 2305 (2.24%), and 102796 (99.95%) genes were aligned to the proteins in the six databases, respectively.

There were 1679 DEGs (1023 upregulated and 656 downregulated) between the fertile and sterile buds. The number of upregulated DEGs in the fertile buds was much higher than the number of downregulated DEGs.

### GO and KEGG Analyses of DEGs

**FIGURE 6:**
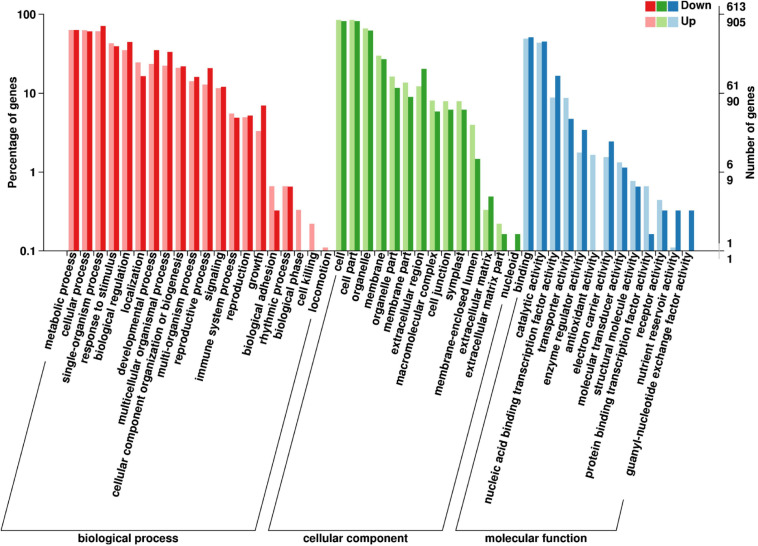
GO annotation of differentially expressed genes (DEGs) between fertile and sterile buds. The *x*-axis shows the terms in the three GO categories, the left *y*-axis shows the percentage of DEGs enriched in a specific term (out of all the DEGs in each category), and the right *y*-axis shows the number of DEGs enriched in a specific term.

Of the 1518 GO annotated DEGs (905 upregulated and 613 downregulated DEGs) in the fertile buds, there were 130, 10, and 69 significantly enriched DEGs (Kolmogorov–Smirnov *P*-value < 0.05) in the GO biological process (BP), cellular component (CC), and molecular function (MF) GO categories, respectively (Supplementary Table 4), comprising 47 functional groups (Figure 6).

In the BP category, the dominant groups were metabolic process (63.04%), cellular process (61.73%), and single-organism process (65.02%). Regarding the most significant term, specification of organ position (GO:0010159), five downregulated DEGs belong to the plant-specific TF YABBY family (Supplementary Figure 7A), which is involved in the feedback regulation of gibberellin biosynthesis in rice (Dai et al., 2007). In the CC category, the dominant groups were cell (83.33%) and cell part (83.33%). Regarding the plant-type cell wall term (GO:0009505), seven aspartic proteinase (AP) and nine pectin methyl-esterase (PME) DEGs were enriched in this term (Supplementary Figure 7A). Aspartic protease participates in stamen development and cell death in rice (Niu et al., 2013; Ko et al., 2014) and PME genes are involved in pollen development and pollen tube growth in *B*. *campestris* (Xiong et al., 2019). In the MF category, the dominant groups were binding (49%) and catalytic activity (44%). Regarding the sugar transmembrane transporter activity term (GO:0051119), six upregulated *SWEET* DEGs in fertile buds were enriched in this term (Supplementary Figure 7A). *SWEET1/8*, which are highly expressed in *Arabidopsis* stamens, play important roles in glucose transport for pollen tube growth and starch accumulation in pollen (Chen et al., 2010).

**FIGURE 7:**
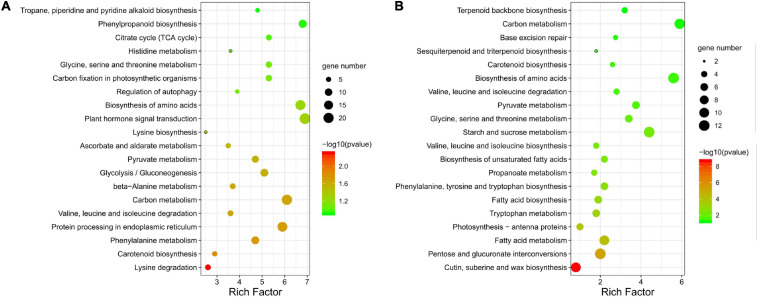
KEGG pathway annotation of differentially expressed genes (DEGs). Top 20 enriched KEGG pathways for **(A)** upregulated and **(B)** downregulated DEGs between fertile and sterile buds. The rich factor is the ratio of the number of DEGs to the total number of genes in each pathway. The color and size of the dots indicate the *P*-value and the number of DEGs mapped to the pathway, respectively.

The 581 KEGG-annotated DEGs (382 upregulated and 199 downregulated) were assigned to 107 KEGG pathways. The five most enriched categories were cellular processes (2.6%), environmental information processing (5.3%), genetic information processing (14.5%), metabolism (76.1%), and organismal systems (1.5%) (Supplementary Table 5). Pollen abortion and fertility restoration were shown to involve transport and catabolism, signal transduction, amino acid metabolism, carbohydrate metabolism, energy metabolism, and metabolism of terpenoids and polyketides, etc. (Supplementary Table 5). More specifically, the upregulated DEGs were significantly enriched in 12 pathways, like lysine degradation (ko00310) and phenylalanine metabolism (ko00360) (Figure 7A). The downregulated DEGs were significantly enriched in 18 pathways, including cutin, suberine, and wax biosynthesis (ko00073), pentose and glucuronate interconversions (ko00040), and fatty acid metabolism (ko01212) (Figure 7B).

### DEGs Specific to Fertile and Sterile Buds

**FIGURE 8:**
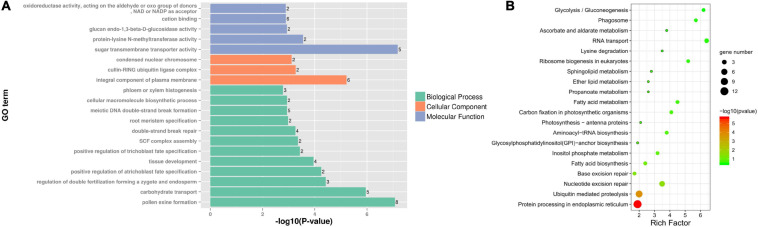
GO and KEGG analyses of differentially expressed genes (DEGs) specific to fertile buds. Enriched **(A)** GO terms and **(B)** KEGG pathways of DEGs specific to fertile buds.

Differentially expressed genes specific to fertile buds may be involved in fertility restoration, while DEGs specific to sterile buds may be associated with CMS. Among the DEGs, 261 specifically expressed genes were identified (9 and 252 in sterile and fertile buds, respectively). GO analysis of the fertile bud-specific DEGs showed that the enriched BP terms included pollen exine formation (GO:0010584), carbohydrate metabolic process (GO:0005975), and SKP1-CUL-F-box (SCF) complex assembly (GO:0010265). The enriched CC terms included integral component of plasma membrane (GO:0005887), cullin-RING ubiquitin ligase complex (GO:0031461), and condensed nuclear chromosome (GO:0000794). Lastly, the enriched MF terms included sugar transmembrane transporter activity (GO:0051119), protein-lysine *N*-methyltransferase activity (GO:0016279), glucan endo-1,3-beta-D-glucosidase activity (GO:0042973), cation binding (GO:0043169), and oxidoreductase activity (acting on the aldehyde or oxo group of donors, NAD or NADP as acceptor, GO:0016620) (Figure 8A).

Additionally, 55 fertile bud-specific DEGs were annotated with KEGG pathways. The four most significant enriched pathways were protein processing in endoplasmic reticulum (ko04141), ubiquitin-mediated proteolysis (ko04120), nucleotide excision repair (ko03420), and base excision repair (ko03410) (Figure 8B).

### DEGs Related to Hormones and Anther/Pollen Development

Stamen development is often modulated by hormone biosynthesis and signaling pathways, and certain hormone-related mutants often display male sterility (Song et al., 2013). We identified 31 hormone-related DEGs. Of these, 3, 9, 7, 4, 1, 2, and 5 were related to gibberellin, auxin, abscisic acid (ABA), cytokinin, ethylene, jasmonic acid (JA) and salicylic acid, respectively (Supplementary Figure 7B). All DEGs related to JA and salicylic acid were upregulated in the fertile compared to the sterile buds. Additionally, *BnaA09g48160D*, which encodes the homologous protein *Arabidopsis* response regulator 4 (ARR4), was specifically expressed in fertile buds. ARR4 modulates photomorphogenesis by interacting with the red/far-red light photoreceptor phytochrome B and regulates the cytokinin response pathway (Mira-Rodado et al., 2007).

After using The *Arabidopsis* Information Resource (TAIR) database to annotate the DEGs, we discovered that 44 DEGs (35 upregulated and 9 downregulated in fertile buds) were related to anther and pollen development (Supplementary Table 6). The upregulated genes included *ALA6*, *UGE3*, *DL1C*, *COX11*, *PSS1*, *ARID1*, *P5CS1*, *MAZ1*, *ABCG6*, *INP1*, *SPS2F*, *KOM*, *UPEX1*, *BHLH010*, *BHLH089*, *MYB108*, *CALS5*, *PRX9*, *PRX40*, *TAP35/TAP44*, *AMS*, *SWEET8*, and *PIRL1*. Among them, *MAZ1*, *ABCG6*, *SPS2F*, *KOM*, and *UPEX1* are involved in pollen wall (including pollen exine) formation. Four *INP1* genes play important roles in pollen surface aperture formation. Two *CALS5* genes (which encode callose synthase 5) are responsible for the synthesis of callose that is deposited at the primary cell wall of meiocytes, tetrads, and microspores. *TAP35/TAP44* and *AMS* participate in tapetal cell development. *SWEET8* and *PIRL1* function in microspore development. Lastly, five *BHLH* and *MYB* TF DEGs (four of which were upregulated) are involved in anther development and dehiscence.

The downregulated genes included *POE1*, *IPE1*, and *BHLH010.* Among them, all seven DEGs of the *POE1* family (which encode Pollen Ole e 1 allergen and extensin family proteins) were downregulated in fertile buds. They can be epigenetically controlled via histone H3 lysine 27 trimethylation (H3K27me3) (Hu et al., 2014).

### PPI Network

The PPI network contained 6077 interactions among 658 proteins, encoded by 423 upregulated, and 235 downregulated DEGs. There were ten modules, which were composed of 610, 18, 9, 5, 3, 3, 3, 3, 2, and 2 DEGs, respectively (Supplementary Figures 8A–J). The 10 hub genes with the top degree scores, were *BnaA09g56400D*, *BnaA10g18210D*, *BnaA10g18220D*, *BnaC09g41740D*, *BnaC04g03080D*, *BnaA05g25330D*, *BnaC03g51080D*, *BnaC09g01600D*, *BnaA01g10430D*, and *BnaCnng18680D*. The first 4 of these had the highest degree scores and were all upregulated in fertile buds (Supplementary Figure 8K). They all encode RAD23d (ubiquitin receptor radiation sensitive23d), which plays essential roles in the cell cycle, morphology, and fertility of higher plants by delivering ubiquitinated substrates to the 26S proteasome (Farmer et al., 2010). *Rfw* may upregulate these *RAD23d* hub genes so that they could modulate the expressions of their interacting genes, thereby mediating fertility restoration. The remaining six hub genes were annotated as *MPK6*, *HAOX2*, *bt1*, and *GSO1* (three times), respectively.

**FIGURE 9:**
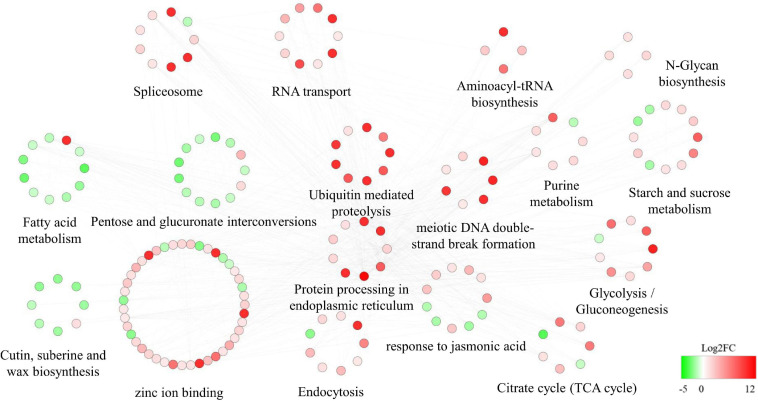
Protein–protein interaction (PPI) network related to the regulator *Rfw*. Red and green indicate upregulated and downregulated differentially expressed genes [DEGs; the shade reflects log2(fold change)].

The DEGs that interacted with the hub genes were subjected to GO and KEGG analyses. Pathways related to carbohydrate metabolism (such as *N*-glycan biosynthesis, glycolysis/gluconeogenesis, the TCA cycle, and starch and sucrose metabolism) tended to be upregulated. The JA response pathway was also upregulated. Other pathways potentially related to male fertility were also dramatically upregulated, such as ubiquitin-mediated proteolysis, purine metabolism, meiotic DNA double-strand break formation, protein processing in endoplasmic reticulum, and endocytosis. In addition, RNA-related pathways (spliceosome, RNA transport, and aminoacyl-tRNA biosynthesis) were upregulated. In contrast, pentose and glucuronate interconversions, fatty acid metabolism, and cutin, suberine, and wax biosynthesis pathways were highly downregulated (Figure 9).

### RT-qPCR Validation

**FIGURE 10:**
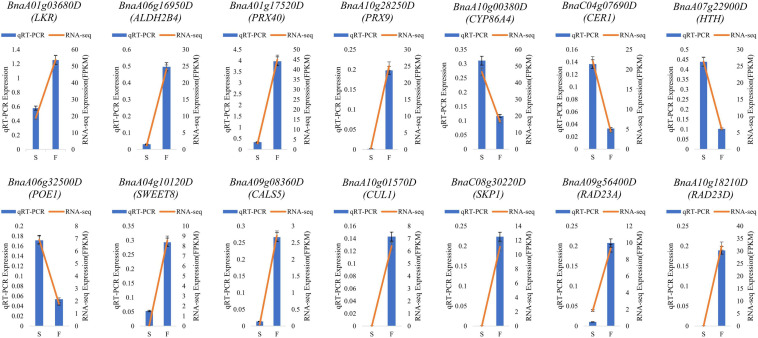
RT-qPCR validation. S, sterile sample; F, fertile sample.

To validate the RNA-seq results, 14 DEGs in enriched pathways and related to fertility restoration were subjected to RT-qPCR (Supplementary Table 7). Of these DEGs, 10 were upregulated in fertile buds, comprising *LKR*, *ALDH2B4*, *PRX40*, *PRX9*, *SWEET8*, *CALS5*, *CUL1*, *SKP1*, *RAD23A*, and *RAD23D*. The remaining four DEGs were downregulated in fertile buds, comprising *CYP86A4*, *CER1*, *HTH*, and *POE1*. The RT-qPCR results showed that their expression tendencies were consistent with those from RNA-seq analysis, indicating the reliability and accuracy of the RNA-seq and the pathway enrichment results (Figure 10).

## Discussion

### Cytological Characteristics and Mitochondrial Gene Transcription in Sterile and Fertile Buds

The *WNJ01A* CMS line displayed a complete pollen abortion phenotype, and its fertility could be restored by the restorer line *Hui01*. By observing paraffin-embedded sections of sterile and fertile buds, we found that the sporogenous cell stage was the critical stage regarding pollen degeneration, with major sporogenous cells exhibiting loose and irregular arrangements in sterile anthers. The pollen abortion stage of *WNJ01A* CMS is earlier than in *pol* CMS and *ogu* CMS. Regarding *pol* CMS, the pollen abortion stage is the polarization stage of the archespore, in which sporogenous cells do not differentiate and normal tetrads are not produced in the sterile anthers (An et al., 2014). Regarding *ogu* CMS, the pollen abortion stage involves the transition from the tetrad to single-nucleus pollen. The pollen development is impaired due to premature death of tapetal cells at the vacuolate microspore stage (Gonzalez-Melendi et al., 2008). Additionally, the pollen-abortion stage of *WNJ01A* CMS is later than that of *hau* CMS; *hau* CMS stamen primordia lack normal polarization and form petal primordia at the differentiation stage of stamen primordia (Wan et al., 2008).

Our RT-qPCR results regarding 32 known protein-coding mitochondrial genes indicate that most of these genes were significantly upregulated in fertile buds. For instance, genes related to mtETC complexes I, III, IV, and V were markedly upregulated in fertile buds. This implies that the mtETC was disrupted (with downregulated expression) in sterile buds. In contrast, in fertile plants, this disruption was prevented, restoring normal pollen development. Certain CMS types also display the male-sterile phenotype due to mtETC disruption (Fujii et al., 2010), such as sugar beet CMS-G (Ducos et al., 2001), pepper CMS-Peterson (Ji et al., 2013), rice CMS-HL (Peng et al., 2010; Wang et al., 2013), sunflower CMS-PET1 (Sabar et al., 2003), and pearl millet CMS plants (Kale and Munjal, 2005). Only one mtETC-related gene (*cob*) was downregulated (non-significantly) in fertile buds.

### Mapping of *Rfw* Gene

*Rfw* was preliminarily mapped to 27.62–33.87 Mb on chromosome 9 by BSA-seq. Interestingly, there are many PPR genes in this region, including two restorer genes, *Rfp* (identified in *Polima* CMS; locus: 31.64–31.67 Mb) (Liu et al., 2012) and *Rfn* (identified in *nap* CMS; locus: 31.79–31.81 Mb) (Liu et al., 2017). Recent research has revealed that a highly dense cluster of restorer-of-fertility-like (*RFL*) genes exists in this region on chromosome A09 and on a collinear region on chromosome C8 in *B. napus* (Ning et al., 2020). A similar *PPR* cluster is present in the collinear region of *Arabidopsis* chromosome A1. The main driving force for evolution of this region may relate to the segmental duplication and retrotransposition processes of A_*n*_ subgenomes (Gaborieau and Brown, 2016). *PPR* genes are probably derived from a few common ancestors and have conserved regulatory roles regarding mitochondrial genes. During evolution, the number of *PPR* genes may have gradually increased and then they may have developed functional specificity (due to sequence variation) related to fertility restoration in relation to various CMS systems.

The *PPR* gene *PPR-Nd1* in the *Hui01* line (which is a homolog of *BnaA09g46030D* in Darmor-bzh) is a candidate *Rfw* gene in the finely mapped *Rfw* interval. *BnaA09g46030D* is known to contain mitochondrial localization signals and is highly upregulated in buds, indicating that it can be regulated and functions in mitochondria (Wang H. et al., 2021). However, comparing the CDS sequences of *PPR-Nd1* and *Rfh* (*BnaA09g46030D*, which restores the sterile phenotype of *hau* CMS, Wang H. et al., 2021), there are multiple SNPs and one InDel (–3 bp) difference, and there are also differences in the amino acid sequences. Critically, whether *PPR-Nd1* can restore the fertility of *WNJ01A* CMS, and the degree of pollen fertility after restoration, remain unknown. In addition, the maintaining and restoring relationship between *WNJ01A* CMS and *Hau* CMS is required to be further analyzed.

### KEGG Analysis of DEGs

In plants, high lysine levels can be toxic to cells (Arruda et al., 2000). For instance, *Arabidopsis* seeds with superabundant lysine exhibited delayed germination and seedling establishment (Zhu and Galili, 2003). Our KEGG analysis of the upregulated DEGs showed that the lysine degradation pathway (ko00310) was enriched, which involved DEGs in the lysine-ketoglutarate reductase/saccharopine dehydrogenase (*LKR*/*SDH*), aldehyde dehydrogenase (*ALDH*), and histone-lysine *N*-methyltransferase/SET domain group (*ASH*/*SDG*) families. These families balance Lys levels in plants (Tang et al., 2000; Stepansky and Galili, 2003).

*LKR/SDH* encode the first two key enzymes in the lysine degradation pathway (Stepansky et al., 2006). They are upregulated in flowers and strongly upregulated by ABA and JA treatment in *Arabidopsis*. ALDHs catalyze oxidation of α-aminoadipic semialdehyde during lysine degradation. Additionally, they are upregulated in *Arabidopsis* anthers and can eliminate lesions induced by toxic aldehydes and reactive oxygen species (Shin et al., 2009; Brocker et al., 2010; Shen et al., 2012; Hou and Bartels, 2015; Zhao et al., 2018). In maize T-CMS, the restorer gene *RF2* encodes an ALDH that can complement the lack of ALDH activity in its mitochondria to reverse the male sterility caused by the CMS protein URF13 (Liu et al., 2001). *ASH/SDG* genes are negative regulators of H3K27me3 (which is known to downregulate genes involved in cellular fate maintenance during development in plants and animals) (Cartagena et al., 2008; Lee et al., 2015; Crevillen, 2020; Cai et al., 2021). *ASH/SDG* is located upstream of *ALDH* in the lysine degradation pathway (ko00310). Hence, *ASH/SDG* probably mediate fertility restoration by affecting the H3K27me3 of the related genes. Our results indicate that upregulation of the lysine degradation pathway is very important to maintain the normal lysine level for anther development in plants with restored fertility.

Another significantly enriched pathway, phenylalanine metabolism (ko00360) involves the peroxidase (*PRX*) and copper amine oxidase (*CuAO*) families. PRX family members, such as PRX9 and PRX40, are essential extensin peroxidases that maintain the integrity of the tapetum and microspore cell walls during anther development in *Arabidopsis* (Jacobowitz et al., 2019). CuAOs mediate plant cell wall formation, maturation, and programmed cell death (Planas-Portell et al., 2013; Tavladoraki et al., 2016). Our results indicate that the phenylalanine metabolic pathway mediates pollen cell wall formation during fertility restoration of *WNJ01A* CMS.

Our results also showed that the downregulated DEGs were enriched in cutin, suberine, and wax biosynthesis (ko00073) and this pathway is involved in normal mature pollen development in *B. napus* (Shi et al., 2020). The DEGs included genes in the *CYP86A*, *CER1-like*, and glucose-methanol-choline (*GMC*) families. *CYP86A* are highly upregulated in mature *Arabidopsis* flowers, and they function as fatty acid ω-hydroxylases (Duan and Schuler, 2005) and may play indispensable roles in pollen tube growth (Koiwai and Matsuzaki, 1988; Wolters-Arts et al., 1998). Cuticular wax is composed of very-long-chain fatty acids (VLCFAs) and VLC alkane biosynthesis is regulated by CER1-like1 specific cofactors (Preuss et al., 1993; Lee and Suh, 2013; Wu et al., 2019). The GMC protein HTH regulates cutin biosynthesis and postgenital organ fusion during flower development (Krolikowski et al., 2003; Xu et al., 2017).

### DEGs Related to Pollen and Anther Development

During the fertility restoration of *WNJ01A* by restorer line *Hui01*, 44 DEGs were confirmed to be involved in pollen and anther development. In fertile buds, multiple genes that play predominant roles in tapetum, pollen wall, and callose development were upregulated. These DEGs included two *bHLH* TFs (*BHLH089* and *BHLH010*), two extensin peroxidases (*PRX9* and *PRX40*), tapetum-specific *TAP35/TAP44*, and two *CALS5* genes. During pollen formation, tapetum cells play central roles in callose degradation, pollen exine formation, and the provision of various nutrients for pollen development (Han et al., 2021). Our cytological characterization of the abortive anthers showed that the tapetum cells (which surround the microspores and provide crucial enzymes and nutrients for microsporogenesis and pollen wall development, Ferguson et al., 2017) were degraded. This likely explains why the microspores were degraded in the sterile buds. Previous studies have highlighted that, during the microspore stage, two *AMS* TFs (Xu et al., 2014), two *SWEET8* (also called *RPG1*) genes (Guan et al., 2008; Chen et al., 2010), *PSS1* (Forsthoefel et al., 2010; Yamaoka et al., 2011), and *P5CS1* (Mattioli et al., 2018) are essential for pollen development. These genes were all upregulated in the fertile buds. In fact, most of the DEGs related to pollen and anther development were upregulated in the fertile buds, indicating that they were modulated by *Rfw* to achieve normal pollen and anther development.

Among the DEGs related to pollen and anther development, three types of genes were significantly downregulated, comprising seven *POE1* (Pollen Ole e 1 allergen and extensin), one *IPE1* (irregular pollen exine1) gene, and one *bHLH10* gene. Recent research has suggested that at least 13 *AtPOE1* gene loci were modified by H3K27me3 in *Arabidopsis* (Hu et al., 2014). Importantly, POE1 protein may participate in pollen tube emergence and guidance (Tang et al., 2002; Alche et al., 2004; Hamman-Khalifa et al., 2008), and H3K27me3 is involved in pollen sporophyte development (Hoffmann and Palmgren, 2013). Regarding *IPE1*, the putative oxidative pathway of ω-hydroxy fatty acids, which depends on *IPE1*, plays significant roles in anther cuticle and pollen exine formation in maize (Chen et al., 2017).

### DEGs Specific to Fertile Buds

Among the KEGG pathways enriched in the DEGs specific to fertile buds, the ubiquitin–proteasome pathway was identified, with three *SKP1-like* genes, three Cullin 1 (*CUL1*) genes, and three *RAD23D* genes being specifically detected in fertile buds. In lily (*Lilium longiflorum*), three *LSK1–3* (*SKP1*-like) genes play critical roles in regulating pollen tube elongation (Chang et al., 2009). In *Arabidopsis*, *AtCUL1* plays an important role in JA signaling (Ren et al., 2005). Furthermore, in *Arabidopsis*, SCF complexes regulate JA-responsive genes related to pollen development (Devoto et al., 2002; Xu et al., 2002). Thus, our results indicate that fertility restoration of *WNJ01A* CMS relies on the ubiquitin-proteasome system (UPS).

### Role of RAD23d in PPI Network

In the PPI network, the hub genes with the highest degree scores were all RAD23d genes. The RAD23 family provides an essential connection between ubiquitylated proteins and the 26S proteasome in *Arabidopsis* (Lahari et al., 2017). In addition, RAD23 has been identified as a ubiquitin-like (UBL)/ubiquitin-associated (UBA) protein, which is key for ubiquitin-mediated protein degradation (Andersson et al., 2005; Zuo and Mahajan, 2005; Dantuma et al., 2009). The UPS is extremely important in plant growth and development as it regulates proteasome-dependent protein turnover (Lowe et al., 2006; Wade and Auble, 2010). During the development of anthers with abnormally active meiosis, DNA repair is essential. Rad protein is involved in recognition of and binding to damaged DNA to correct DNA lesions (Gallego et al., 2000). Rad23 can also efficiently initiate the cell cycle and nucleotide excision repair-like repair pathway (Lahari et al., 2017; Okeke et al., 2020). Interestingly, the UPS not only removes incorrectly folded proteins, but also ensures appropriate spatial and temporal protein distributions (Medina et al., 2012; Liang et al., 2014; Chen and Walters, 2016). For example, RAD23B is key in pollen development as it controls the turnover of the key cell cycle protein KIP-related protein 1 (KRP1) (Li et al., 2020). In summary, RAD23d proteins may mediate fertility restoration of *WNJ01A*, regulated by *Rfw*, by repairing damaged DNA and degrading sterility-related proteins via the UPS, along with potentially regulating the degradation of other proteins and modulating hormone signals.

### Mechanistic Model

**FIGURE 11:**
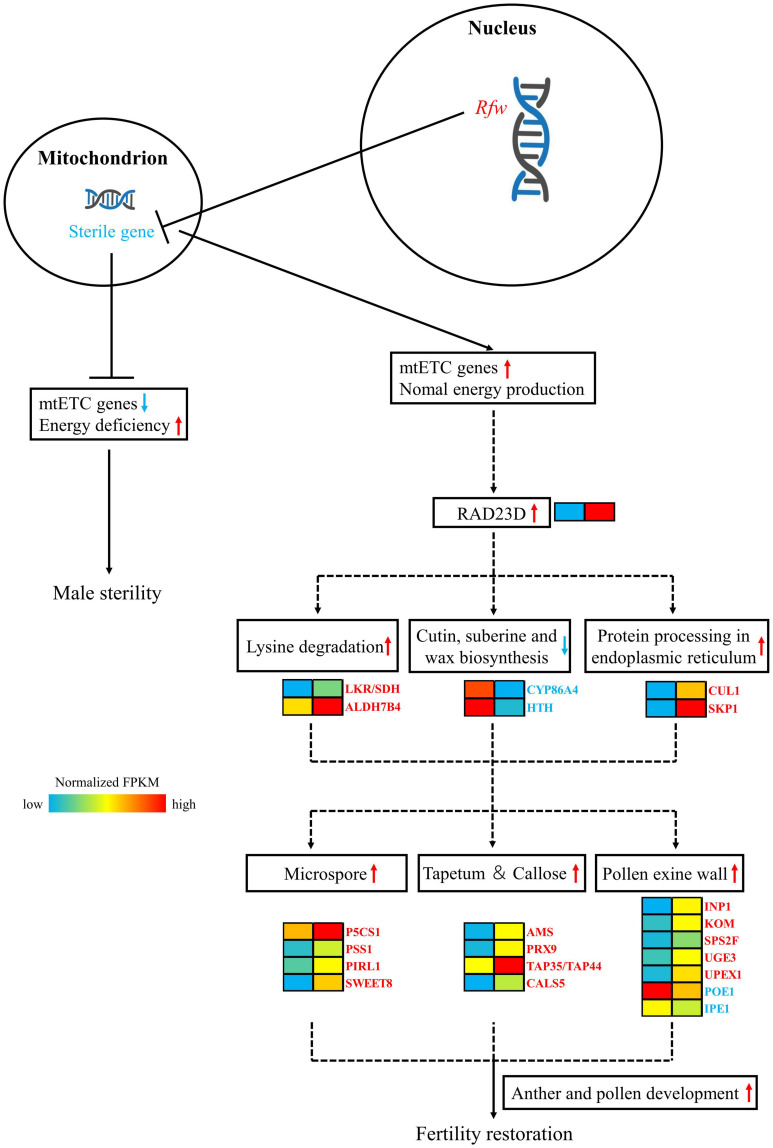
Mechanistic model of the role of the sterile gene of *WNJ01A* CMS and the role of *Rfw* in fertility restoration. In the heat map, the left-hand side represents sterile buds and the right-hand side represents fertile buds.

After transcriptome sequencing of sterile and fertile flower buds, the fertile/sterile bud-specific DEGs, pollen/anther development- and hormone-related DEGs, and DEGs in the PPI network were subjected to GO and KEGG analysis. Based on the results, we propose a model of the mechanism of fertility restoration regulated by the restorer gene *Rfw* (Figure 11).

In mitochondria, the male sterility gene downregulates mtETC genes, which causes energy deficiency and results in the formation of flower buds exhibiting male sterility. The nuclear genome possesses an *Rfw* restorer gene, which can inhibit the expression of the male sterility gene at the level of transcription or translation or by another mechanism. The *Rfw* gene ensures that the function of the mtETC genes and mitochondrial energy production return to normal.

As a result of the effect of *Rfw*, *RAD23* might be initially up-regulated in fertile buds. Then, the genes *LKR/SDH*, *ALDH7B4*, *CUL1*, *SKP1* and their related pathways of lysine degradation, protein processing in endoplasmic reticulum are upregulated. In contrast, the genes *CYP86A*, *HTH* and their related pathways of cutin, suberine, and wax biosynthesis are downregulated. Later, the genes related to tapetum, pollen exine wall, microspore, and callose development, such as *SWEET8*, *CALS5*, *TAP35/44*, *PRX9*, *PRX40*, *AMS*, *etc.* are upregulated and *POE1*, *IPE1*, *etc*. are downregulated. Ultimately, the biological processes of anther and pollen development exhibit normal function and the flower buds restored fertility.

## Data Availability Statement

The datasets presented in this study can be found in online repositories. The names of the repository/repositories and accession number(s) can be found below: NCBI BioProject, PRJNA739480.

## Author Contributions

CL and QY conceived the experiment. FW, CZ, and JW provided advice on the experimental design. QY performed most of the experiments and analyzed the data. XN participated in paraffin-embedded sections experiments. JX participated in phenotypic investigate. FH participated in data analysis. CL and QY wrote the manuscript. All the authors reviewed and approved this submission.

## Conflict of Interest

The authors declare that the research was conducted in the absence of any commercial or financial relationships that could be construed as a potential conflict of interest. The reviewer ZL declared a shared affiliation, with no collaboration, with the authors to the handling editor at the time of the review.

## Publisher’s Note

All claims expressed in this article are solely those of the authors and do not necessarily represent those of their affiliated organizations, or those of the publisher, the editors and the reviewers. Any product that may be evaluated in this article, or claim that may be made by its manufacturer, is not guaranteed or endorsed by the publisher.

## Footnotes

1
http://www.softberry.com/

2
http://www.bioinformatics.com.cn/

3
http://string-db.org/

## Supplementary Material

Supplementary Figure 1 Two sequenced BAC clones (four replicates each) verified using molecular markers. The closest flanking molecular markers (ID-595A and SR30) and a co-segregating marker (TY21) in the finely mapped candidate interval were identified by PCR amplification. PCR products were detected using 1.2% agarose gel.

Supplementary Figure 2 Full-length CDS region alignment between PPR-Nd1, BnaA09g46030D, BjuA044087, and Bra026882. Black background: consensus sequences; red line: PPR motif region.

Supplementary Figure 3 Full-length protein region alignment between PPR-Nd1, BnaA09g46030D, BjuA044087, and Bra026882. Black background: consensus sequences; red line: PPR motif region; purple line: motif I; blue line: motif II.

Supplementary Figure 4 Full-length CDS region alignment between PPR-Nd2, BjuA044085, and Bra026884. Black background: consensus sequences; red line: PPR motif region.

Supplementary Figure 5 Full-length protein region alignment between PPR-Nd2, BjuA044085 and Bra026884. Black background: consensus sequences; red line: PPR motif region.

Supplementary Figure 6 Pearson correlation analysis of the transcript levels in three fertile plant buds (*RFW1*, *RFW2*, and *RFW3*) and three sterile plant buds (*rfw1*, *rfw2*, and *rfw3*).

Supplementary Figure 7 Heatmap of DEGs. **(A)** *YABBY*, *PEM*, *APs*, and *SWEET* family differentially expressed genes (between fertile and sterile buds) that were enriched in the top ten GO terms. BP, biological process; CC, cellular component; MF, molecular function. **(B)** Plant hormone-related genes that were differentially expressed between fertile and sterile buds. GA, gibberellin; IAA, auxin; ABA, abscisic acid; CTK, cytokinin; ET, ethylene; JA, jasmonic acid; SA, salicylic acid.

Supplementary Figure 8 Protein–protein interaction (PPI) network analysis of DEGs. **(A–J)** Ten modules in PPI network. **(K)** Heatmap of four hub genes.

Supplementary Table 1 Primers used in this study.

Supplementary Table 2 Primers for 32 known protein-coding genes.

Supplementary Table 3 Gene annotation of 99.75 kb DNA fragment.

Supplementary Table 4 GO annotation of DEGs.

Supplementary Table 5 KEGG pathway annotation of DEGs.

Supplementary Table 6 DEGs related to pollen and anther development.

Supplementary Table 7 DEGs selected for RT-qPCR verification.
