# Supplementary figures and images for: Unraveling the Genetic Basis of Fertility Restoration for Cytoplasmic Male Sterile Line WNJ01A Originated From Brassica juncea in Brassica napus
Source: Front Plant Sci. 2021 Aug 31;12:721980. doi: 10.3389/fpls.2021.721980 (PMC8438535; doi:10.3389/fpls.2021.721980)

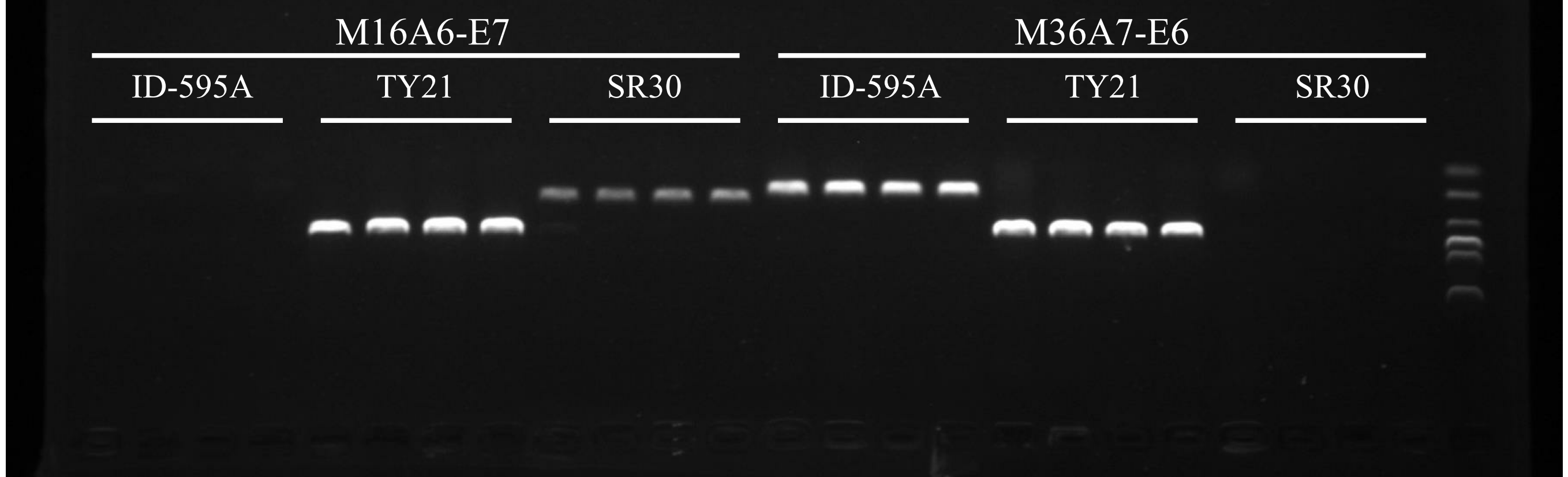

Supplement: Supplementary Figure 1 — Two sequenced BAC clones (four replicates each) verified using molecular markers. The closest flanking molecular markers (ID-595A and SR30) and a co-segregating marker (TY21) in the finely mapped candidate interval were identified by PCR amplification. PCR products were detected using 1.2% agarose gel. [file Image_1.TIF]

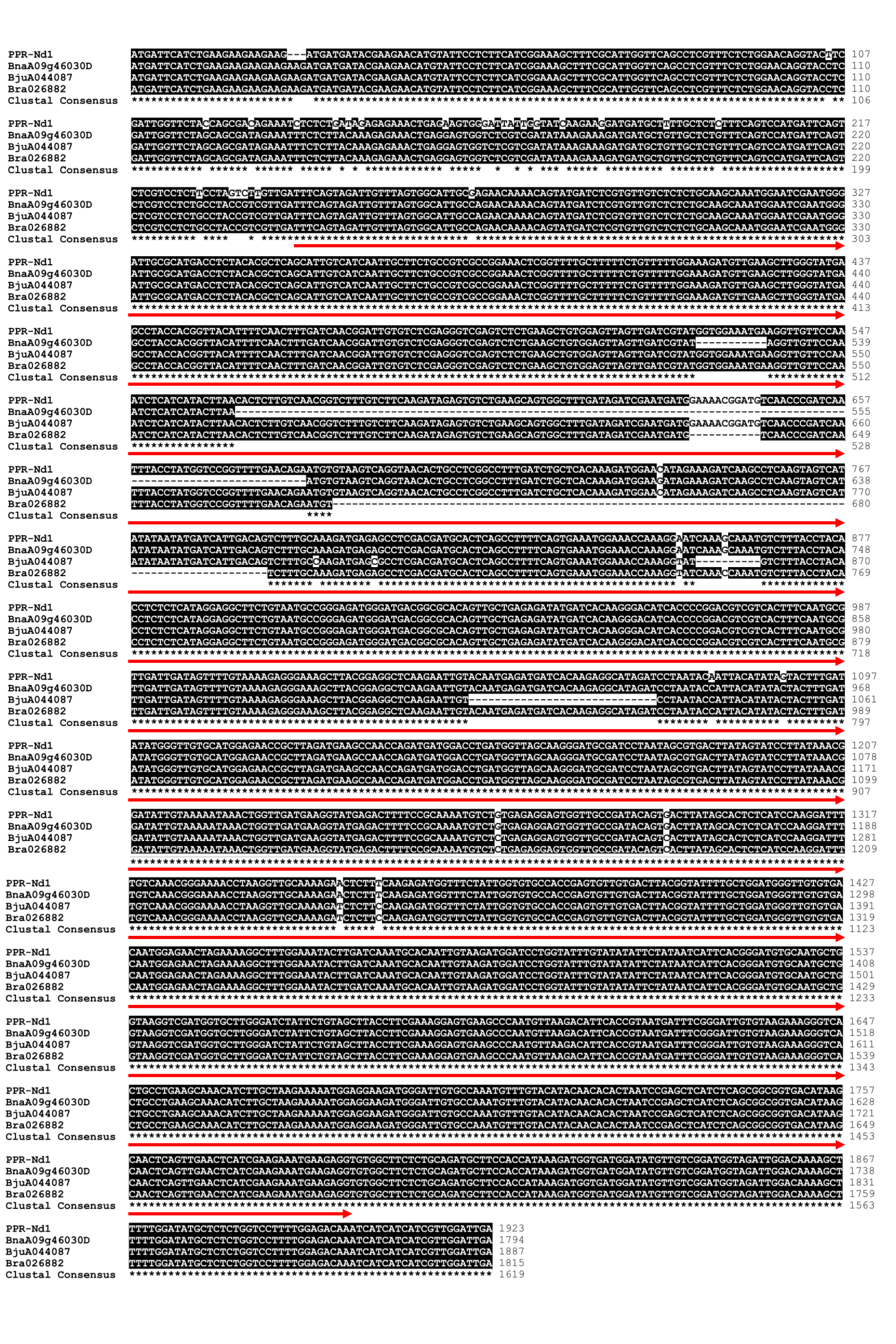

Supplement: Supplementary Figure 2 — Full-length CDS region alignment between PPR-Nd1, BnaA09g46030D, BjuA044087, and Bra026882. Black background: consensus sequences; red line: PPR motif region. [file Image_2.TIF]

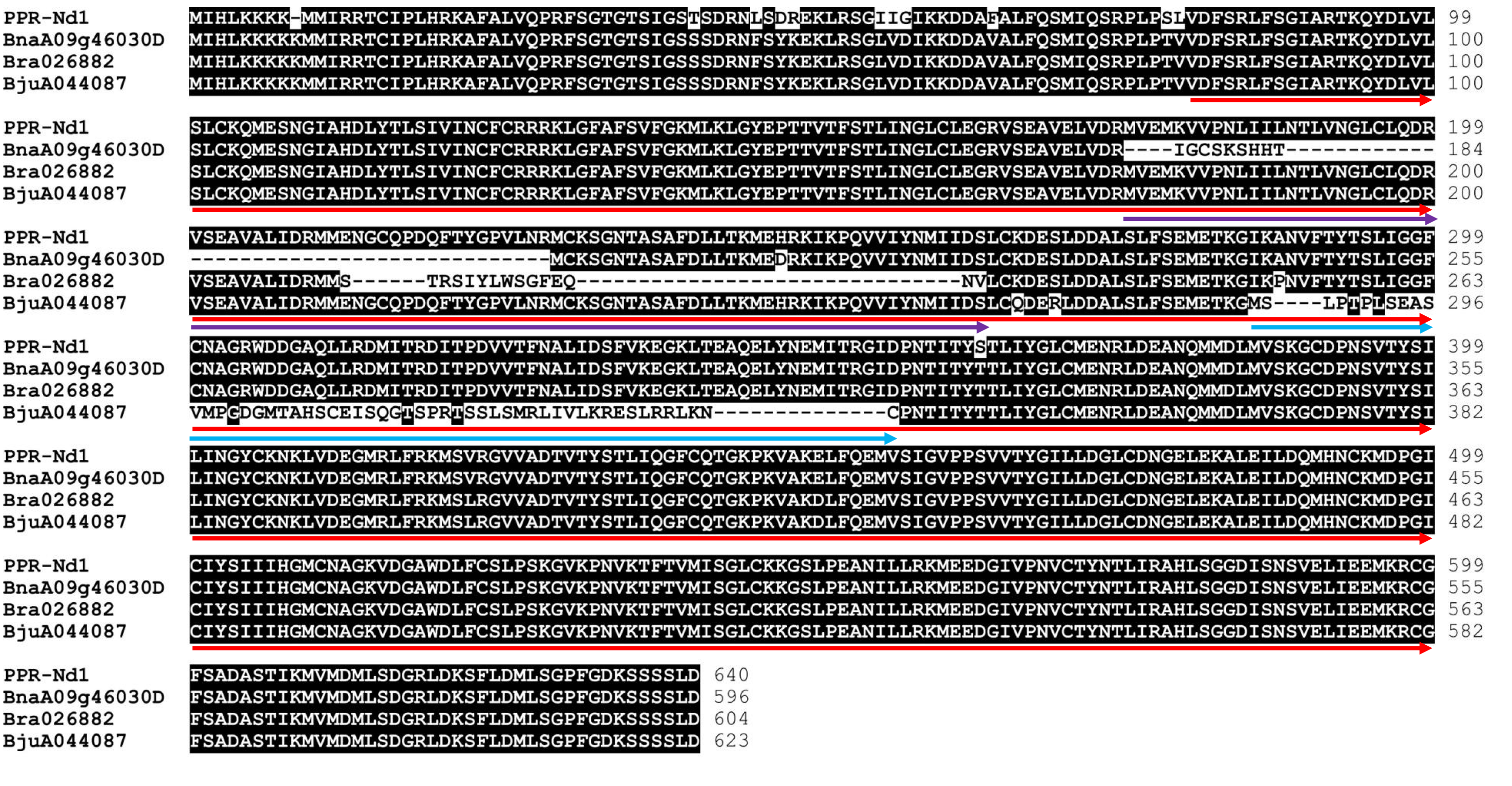

Supplement: Supplementary Figure 3 — Full-length protein region alignment between PPR-Nd1, BnaA09g46030D, BjuA044087, and Bra026882. Black background: consensus sequences; red line: PPR motif region; purple line: motif I; blue line: motif II. [file Image_3.TIF]

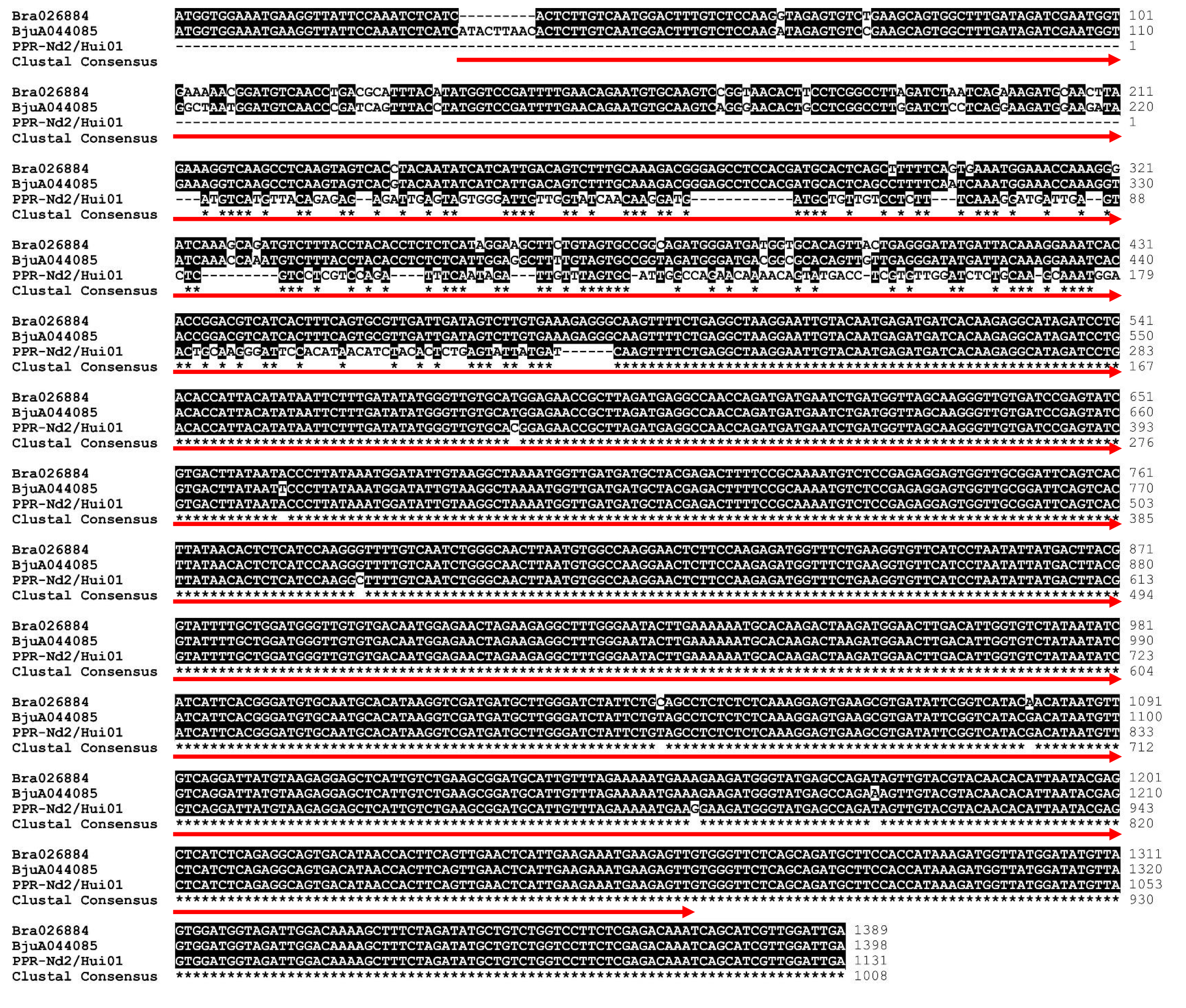

Supplement: Supplementary Figure 4 — Full-length CDS region alignment between PPR-Nd2, BjuA044085, and Bra026884. Black background: consensus sequences; red line: PPR motif region. [file Image_4.TIF]

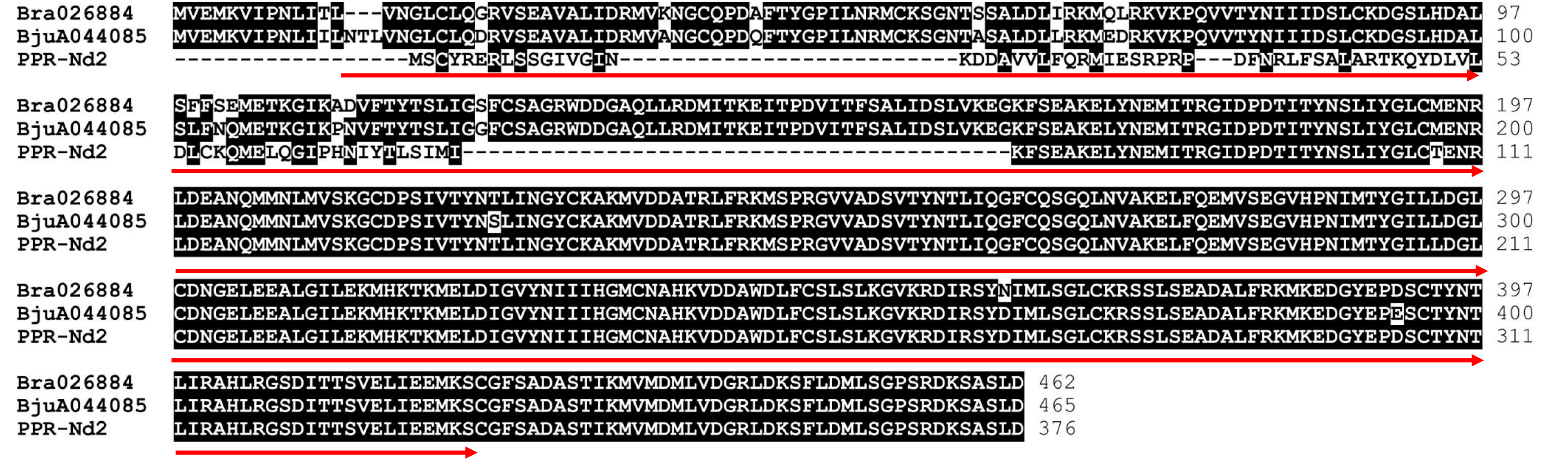

Supplement: Supplementary Figure 5 — Full-length protein region alignment between PPR-Nd2, BjuA044085 and Bra026884. Black background: consensus sequences; red line: PPR motif region. [file Image_5.TIF]

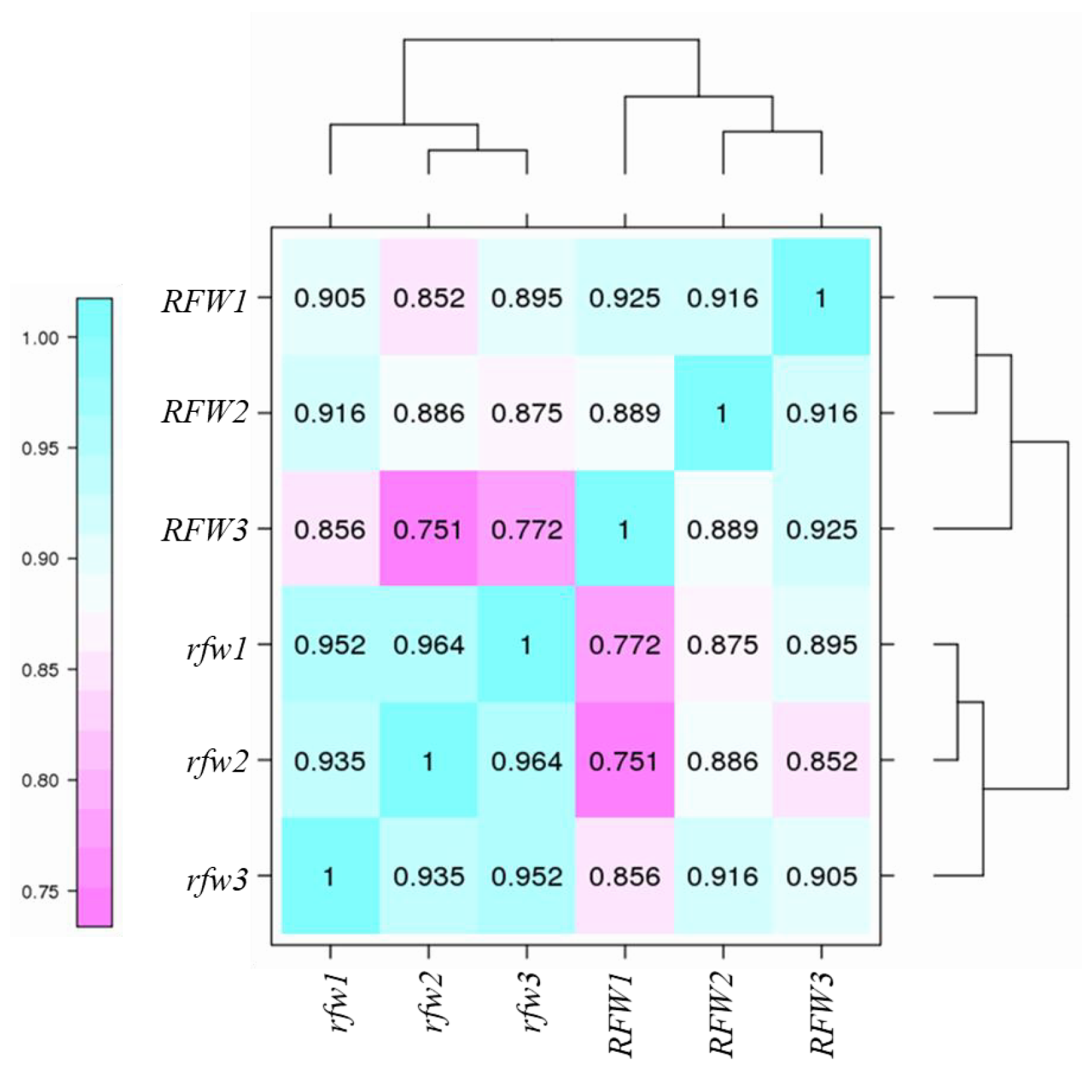

Supplement: Supplementary Figure 6 — Pearson correlation analysis of the transcript levels in three fertile plant buds (RFW1, RFW2, and RFW3) and three sterile plant buds (rfw1, rfw2, and rfw3). [file Image_6.TIF]

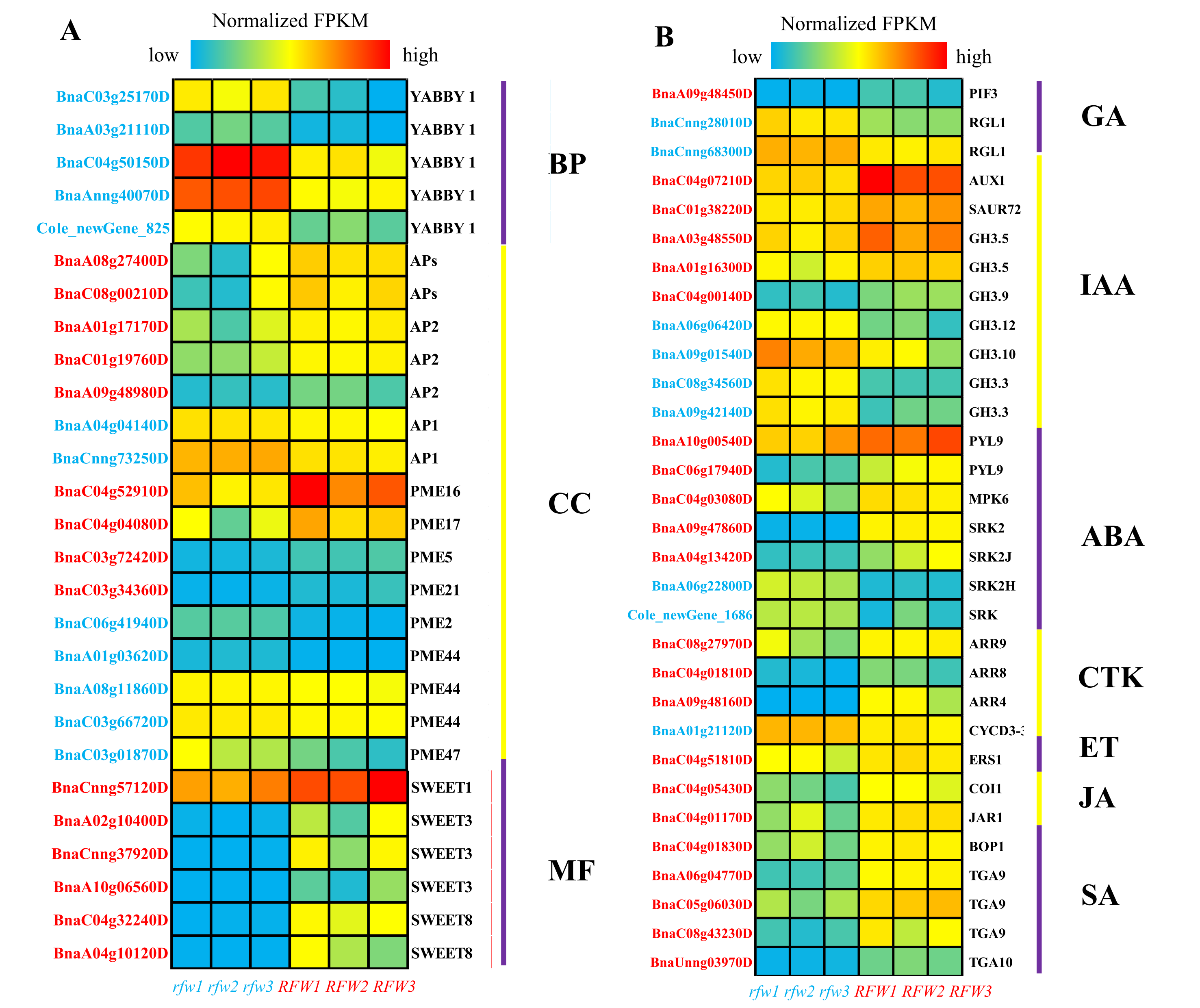

Supplement: Supplementary Figure 7 — Heatmap of DEGs. (A) YABBY, PEM, APs, and SWEET family differentially expressed genes (between fertile and sterile buds) that were enriched in the top ten GO terms. BP, biological process; CC, cellular component; MF, molecular function. (B) Plant hormone-related genes that were differentially expressed between fertile and sterile buds. GA, gibberellin; IAA, auxin; ABA, abscisic acid; CTK, cytokinin; ET, ethylene; JA, jasmonic acid; SA, salicylic acid. [file Image_7.TIF]

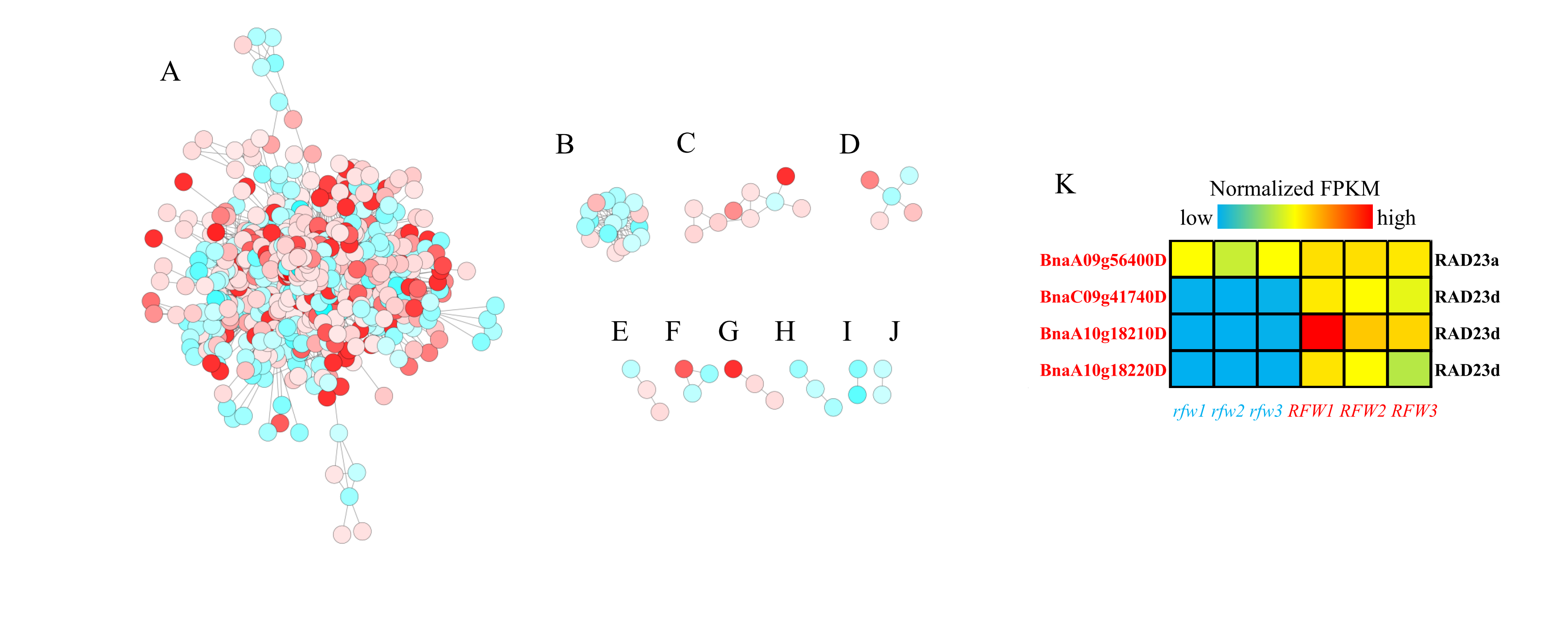

Supplement: Supplementary Figure 8 — Protein–protein interaction (PPI) network analysis of DEGs. (A–J) Ten modules in PPI network. (K) Heatmap of four hub genes. [file Image_8.TIF]
